# Prenatal exposure to antiseizure medications and fetal growth: a population-based cohort study from the Nordic countries

**DOI:** 10.1016/j.lanepe.2024.100849

**Published:** 2024-02-08

**Authors:** Jakob Christensen, Helga Zoega, Maarit K. Leinonen, Nils Erik Gilhus, Mika Gissler, Jannicke Igland, Yuelian Sun, Torbjörn Tomson, Silje Alvestad, Marte-Helene Bjørk, Julie Werenberg Dreier

**Affiliations:** a Department of Neurology, Affiliated Member of the European Reference Network EpiCARE, Aarhus University Hospital, Aarhus, Denmark; b Department of Clinical Medicine, Aarhus University, Aarhus, Denmark; c Centre of Public Health Sciences, Faculty of Medicine, University of Iceland, Reykjavik, Iceland; d School of Population Health, Faculty of Medicine and Health, UNSW Sydney, Sydney, Australia; e Department of Knowledge Brokers, Finnish Institute for Health and Welfare, Helsinki, Finland; f Department of Clinical Medicine, University of Bergen, Bergen, Norway; g Department of Neurology, Haukeland University Hospital, Bergen, Norway; h Region Stockholm, Academic Primary Health Care Centre, Stockholm, Sweden; i Department of Global Public Health and Primary Care, University of Bergen, Bergen, Norway; j Department of Health and Caring Sciences, Western Norway University of Applied Sciences, Bergen, Norway; k Centre for Integrated Register-based Research, CIRRAU, Aarhus University, Aarhus, Denmark; l Department of Clinical Epidemiology, Aarhus University Hospital, Aarhus, Denmark; m Department of Clinical Neuroscience, Karolinska Institutet, Stockholm, Sweden; n National Center for Epilepsy, Oslo University Hospital, Oslo, Norway; o National Centre for Register-based Research, School of Business and Social Sciences, Aarhus University, Denmark; p Karolinska Institute, Department of Molecular Medicine and Surgery, Stockholm, Sweden

**Keywords:** Birth weight, Head circumference, Gestational age, In utero, Pregnancy, Antiepileptic drugs, Small for gestational age

## Abstract

**Background:**

The short- and long-term consequences of restricted fetal growth cause considerable concern, and how prenatal exposure to different antiseizure medications (ASMs) affects fetal growth remains uncertain.

**Methods:**

This was a population-based cohort study of liveborn singleton children born in Denmark, Finland, Iceland, Norway, and Sweden from 1996 to 2017. Prenatal exposure was defined as maternal filling of prescriptions for ASM during pregnancy registered in national prescription registries and primary outcomes were adjusted odds ratios (aORs) of microcephaly or being born small for gestational age.

**Findings:**

We identified 4,494,918 children (males: 51.3%, 2,306,991/4,494,918), including 38,714 (0.9%) children of mothers with epilepsy. In the overall population, prenatal monotherapy exposure with carbamazepine (aOR: 1.25 (95% CI: 1.12–1.40)), pregabalin (aOR: 1.16 (95% CI: 1.02–1.31)), oxcarbazepine (aOR: 1.48 (95% CI: 1.28–1.71)), clonazepam (aOR: 1.27 (95% CI: 1.10–1.48)), and topiramate (aOR: 1.48 (95% CI: 1.18–1.85)) was associated with risk of being born small for gestational age, and carbamazepine was associated with microcephaly (aOR: 1.43 (95% CI: 1.17–1.75)). In children of mothers with epilepsy, prenatal exposure to carbamazepine (aOR: 1.27 (95% CI: 1.11–1.47)), oxcarbazepine (aOR: 1.42 (95% CI: 1.18–1.70)), clonazepam (aOR: 1.40 (95% CI: 1.03–1.89)), and topiramate (aOR: 1.86 (95% CI: 1.36–2.54)) was associated with being born small for gestational age; carbamazepine, with microcephaly (aOR: 1.51 (95% CI: 1.17–1.95)). No associations with small for gestational age and microcephaly were identified after prenatal exposure to lamotrigine, valproate, gabapentin, levetiracetam, phenobarbital, acetazolamide, phenytoin, clobazam, primidone, zonisamide, vigabatrin, ethosuximide and lacosamide, but except for lamotrigine, valproate, gabapentin, and levetiracetam, numbers of exposed children were small.

**Interpretation:**

Prenatal exposure to carbamazepine, oxcarbazepine, clonazepam, and topiramate was associated with increased risk of being born small for gestational age in both the overall population and in children of women with epilepsy suggesting that prenatal exposure to these drugs is associated with fetal growth restriction.

**Funding:**

The NordForsk Nordic Program on Health and Welfare (83539), the 10.13039/501100004836Independent Research Fund Denmark (1133-00026B), the Danish Epilepsy Association, the Central Denmark Region, the 10.13039/501100009708Novo Nordisk Foundation (NNF16OC0019126 and NNF22OC0075033), and the 10.13039/501100003554Lundbeck Foundation (R400-2022-1205).

**Research in context**

**Evidence before this study**

We searched PubMed for articles published between 1 January 2000 and 31 December 2022 that describe birth weight and head circumference in children prenatally exposed to antiseizure medication. The included search terms were (“anticonvulsants” [MeSH Terms] OR “anticonvulsants” [Text Word]) AND (“pregnancy" [MeSH Terms] OR pregnancy [Text Word]) AND ((“birth weight" [MeSH Terms] OR birth weight [Text Word]) OR (“microcephaly" [MeSH Terms] OR microcephaly [Text Word]) OR head circumference [Text Word]). We identified numerous reports describing exposure to antiseizure medications in pregnancy and associations with indicators of fetal growth restriction, including small for gestational age. A comprehensive review assessing fetal growth reported that use of phenobarbital, topiramate, and zonisamide, but not lamotrigine and levetiracetam during pregnancy, was associated with increased risk of having a baby born small for its gestational age. For all other antiseizure medications commonly used in pregnancy, the data were either too limited or inconsistent. Limited evidence was available to assess head circumference and risk of microcephaly associated with prenatal exposure to specific antiseizure medications, but one study reported an association between prenatal exposure to clonazepam and microcephaly, and three individual studies reported reduced fetal head circumference after prenatal carbamazepine exposure. Thus, the evidence on the potentially harmful effects of some antiseizure medications on fetal growth remains inconclusive.

**Added value of this study**

In this population-based cohort study of more than 4 million liveborn singleton children born in the Nordic Countries, prenatal exposure to carbamazepine, oxcarbazepine, clonazepam, and topiramate was associated with an increased risk of being born small for gestational age in the overall population and in children of women with epilepsy suggesting that prenatal exposure to these drugs is associated with fetal growth restriction. Lamotrigine, the most frequently used antiseizure medication in pregnancy, was not associated with risk of being born *small for gestational age*. Prenatal exposure to carbamazepine was the only antiseizure medication consistently associated with increased risk of being born with microcephaly.

**Implications of all the available evidence**

Our findings raise concern about risk of fetal growth restriction associated with several specific antiseizure medications used in pregnancy.

## Introduction

Fetal growth restriction represents a severe pregnancy complication associated with short- and long-term adverse health outcomes.^1^^,^^2^ Exposure to antiseizure medications (ASMs) in pregnancy has been associated with indicators of fetal growth restriction, including small for gestational age (SGA)^3^ and microcephaly.^4^, ^5^, ^6^, ^7^ A recently published review reported that pregnancy use of phenobarbital, topiramate, and zonisamide, but not lamotrigine and levetiracetam, was associated with increased risk of having a baby born small for its gestational age.^4^ For all other ASMs commonly used in pregnancy, the data were either too limited or inconsistent.^4^ Limited evidence was available to assess the risk of microcephaly associated with prenatal exposure to specific ASMs,^4^ but one study reported an association between clonazepam and microcephaly,^8^ and three individual studies reported reduced fetal head circumference after prenatal carbamazepine exposure.^6^^,^^9^^,^^10^ Chemically, ASMs are heterogeneous drugs that are both related (e.g., oxcarbazepine and carbamazepine) and unrelated (e.g., topiramate and clonazepam).^11^ However, in animal studies, prenatal exposure to carbamazepine,^12^^,^^13^ oxarbazepine,^14^ clonazepam^15^ and topiramate^16^ have all been associated with reduced fetal growth. Thus, although animal studies suggest potential harm, the human evidence of some ASMs remains inconclusive.^4^ Few studies have had sufficient power to assess the risk associated with prenatal exposure to ASM and make firm conclusions regarding fetal growth.

Given concern for the short- and long-term consequences of restricted intrauterine growth^1^^,^^2^ and in light of reports indicating that prenatal exposure to some ASMs is associated with fetal growth restriction,^4^, ^5^, ^6^, ^7^^,^^10^ we examined the association between prenatal ASM exposure and the risks of fetal growth restriction in a dataset comprising more than four million pregnancies. We assessed several key measures of fetal growth restriction including absolute reductions in birth weight and head circumference and relative risks of being born with a low birth weight (<2500 g), SGA, and microcephaly.

## Methods

### Study design, setting, and population

This was a Nordic population-based cohort study including register data from Denmark, Finland, Iceland, Norway, and Sweden–the SCAN-AED project (www.scanaed.org). Individual-level data from the nationwide health and social registers from these five countries were merged and harmonized. Personal identification numbers are issued to citizens in each of the Nordic countries. The identification number is unique to each person and was used to link information across national registers in each of the countries.

The cohort included all live-born singletons born in Denmark (1997–2017), Finland (1996–2016), Iceland (2004–2017), Norway (2005–2017), and Sweden (2006–2017), Fig. 1. For these years, all included registers had full population coverage. An overview of the data sources and the variables used is presented in Table S1a and b. We excluded a total of 43,208 (1.0%) children due to missing or invalid birth weight and/or gestational age measures; and 8472 (0.2%) children, due to chromosomal abnormalities. We assessed growth measures in all included children in the entire population and in the sub-population of children of mothers with epilepsy. Epilepsy in the mother was defined by a hospital contact with epilepsy before giving birth (International Statistical Classification of Diseases and Related Health Problems, 10th revision (ICD-10) G40-G41, all countries), use of ASM with epilepsy as indication or reason for reimbursement before time of birth (Denmark since 2004, Norway and Finland), or any registered diagnosis of epilepsy in the Medical Birth Register (all countries except Denmark).^17^

**Fig. 1:**
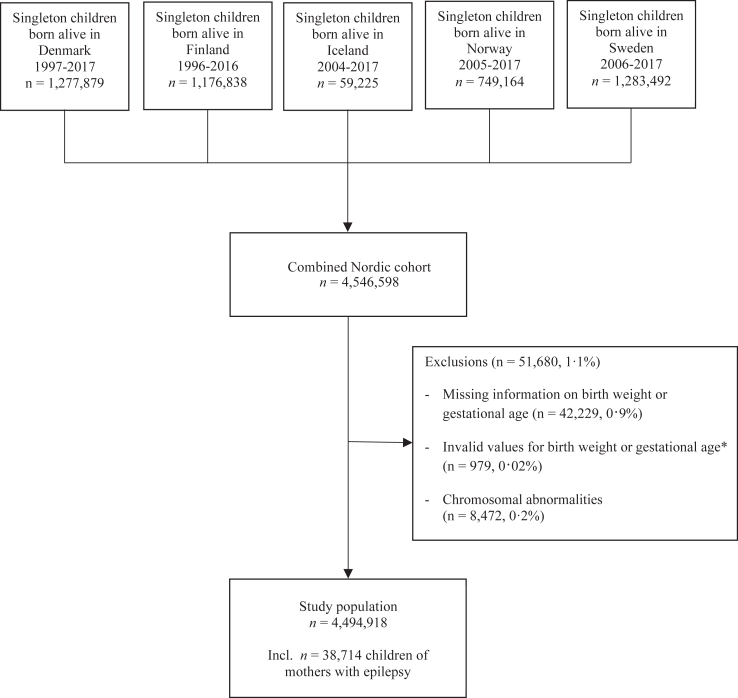
Flowchart of the study population of live-born singleton children in Denmark, Finland, Iceland, Norway, and Sweden. ∗Invalid values were defined as birth weight <300 g or >7000 g; gestational week <22 or ≥45; or if the country- and sex-specific *z*-score for birth weight >4 in children with gestational week <35.

### Prenatal exposure to ASM

We defined prenatal exposure to ASM as maternal filling of ASM prescriptions during pregnancy identified via national prescription registers. These registers contain the Anatomical Therapeutic Chemical (ATC) classification code (www.whocc.no) and the date of dispensing.^18^ Children of mothers who had redeemed ≥1 ASM prescription (ATC codes: N03A (antiseizure medications), N05BA09 (clobazam), or S01EC01 (acetazolamide, data not available in Finland)) from 30 days before the first day of the last menstrual period to the date of birth (i.e., the exposure period) were considered prenatally exposed. The date of the first day of the last menstrual period was estimated by subtracting gestational age at birth (in days) from the date of birth as recorded in the national birth registers. The gestational age at birth was primarily determined by ultrasound. We defined ASM monotherapy as having redeemed one or more prescriptions for a specific ASM and no prescriptions for any other ASM in the exposure period. We defined polytherapy as having redeemed one or more prescriptions for at least two different ASMs during the exposure period. The estimated mean daily dose of ASM was calculated from the total amount of ASMs filled during the time from 30 days before pregnancy to birth divided by the number of days in the same period. The defined daily dose (DDD) is the assumed average maintenance dose per day for a drug used for the main indication of the drug in adults (https://www.who.int/tools/atc-ddd-toolkit/about-ddd). Using the DDD, we dichotomized the estimated daily ASM dose into high (≥50% DDD) and low (<50% DDD).

### Birth characteristics

Information on birth weight, gestational age and head circumference at birth was obtained from the medical birth registers for all births occurring at 22 weeks’ gestation or later. We considered the following measures of fetal growth: birth weight as a continuous measure in grams (g), birth weight as a dichotomized measure (low birth weight; <2500 g), and whether the child was born small for its gestational age, defined as a *z*-score for birth weight ≤10th percentile for gestational week, sex, and country (also including newborns with congenital malformations). Because we have nationwide data (i.e., all children born in the countries in the periods in question), we estimated the z-scores and corresponding percentile cutoffs directly from the distribution of values in our own data. It has recently been suggested that birth weight less than the 3rd percentile is a more specific indicator of fetal growth restriction compared to the 10th percentile.^19^ In additional analyses, we therefore estimated whether the child was born small for its gestational age, defined as a *z*-score for birth weight ≤3rd percentile.^19^ We assessed head circumference as a continuous measure in centimeters (cm) and defined microcephaly as a *z*-score for head circumference ≤3rd percentile for gestational week, sex, and country (also including newborns with congenital malformations).^20^ For children with missing (n = 521,562, 11.6%) or unlikely head circumference values (<15 cm or >45 cm; n = 1735, <0.1%), we estimated an imputed value using multiple imputation by chained equations (MICE). For the imputation model, we included information on gestational age at birth, birth weight, and other important predictors of head circumference (eMethods in the Supplement). Microcephaly was defined for each of the 20 imputed datasets using the combined distribution of observed and imputed values for head circumference.^21^ We applied the approach “Impute, then transform”–where missing values of head circumference is imputed by the model and microcephaly is afterwards estimated using the observed or imputed value of head circumference. We chose this method (instead of also imputing microcephaly), to ensure consistency between head circumference and microcephaly within each of the 20 imputed datasets.

### Statistical analysis

We used linear regression models to estimate the mean difference in birth weight and head circumference and corresponding 95% confidence intervals (CIs) between children with and without prenatal exposure to specific ASMs in monotherapy. We used logistic regression models to estimate odds ratios (ORs) for the association of ASM and ASM dose in pregnancy with low birth weight, SGA, and microcephaly. Confidence intervals were computed with nonclustered standard errors. Potential confounders were selected based on a priori knowledge and all models were adjusted for country of birth, year of birth, sex of child, maternal age, maternal parity, maternal marital or cohabitation status, pre-pregnancy hospital admittances, maternal education, smoking in early pregnancy (yes/no), maternal psychiatric history (ICD-10: codes F00-F99) (yes/no), and use of psychotropic drugs in pregnancy (ATC codes: N06A, N05A, N05B, excl. N05BA09 clobazam) (yes/no). In the full population analysis, but not in the analysis restricted to children of mothers with epilepsy, we adjusted estimates for maternal epilepsy. We imputed missing data for variables with >2% missing (i.e., maternal education, smoking, and head circumference as described previously) with MICE (eMethods in the Supplement). All analyses were conducted separately for the full population first and then for the subpopulation of children of mothers with epilepsy.

Estimates were not adjusted for multiple comparisons and the findings of this study may thus be considered exploratory as priority was given to reduce the probability of producing false negative associations. The findings of this study should thus be replicated in other studies of prenatal exposure to ASMs.

In supplementary analyses, we analyzed the association of prenatal exposure to ASM including polytherapy with low birth weight, SGA, and microcephaly. By not restricting to monotherapy exposure in these analyses, we increased the number of exposed children, which allowed for analyses of more rarely used ASMs.

To make identification of maternal epilepsy equal across all countries, we performed sensitivity analyses where we defined “active” epilepsy in the mother as a diagnosis of epilepsy in the Medical Birth Register, filling a prescription for ASM with the indication “epilepsy” or a hospital contact with a diagnosis of epilepsy within one year of the last menstrual period.

The main analyses were analyzed using imputed data, but we performed complete case analyses (i.e., excluding cases with missing information on head circumference, maternal smoking, and maternal education) to allow comparison of estimates based on the two approaches. We analyzed SGA and microcephaly in children exposed and unexposed to ASM in monotherapy during pregnancy after restricting the exposure period to the period from the first day of the last menstrual period to birth, and head circumference and microcephaly after excluding children with major congenital malformations diagnosed within 1 year of birth and recorded in the medical birth, patient, malformation, or death register as defined by Cohen et al.^22^

To account for confounding by indication, we performed sensitivity analyses where we used prenatal exposure to lamotrigine as an active comparator.

Finally, we estimated the risk of SGA and microcephaly in 3,041,503 children with information of maternal body mass index (BMI) because maternal BMI may influence the choice of ASM as well as fetal growth.

We present results on a given ASM when at least five exposed children with the outcome of interest were identified.

We performed statistical analyses using Stata, version 16.

### Study approvals

The relevant ethical and/or data protection authorities in all countries approved the project (Table S1c).

### Role of the funding source

The funder had no role in the study design, data collection, data analysis, data interpretation, or drafting of the report.

## Results

**Fig. 2:**
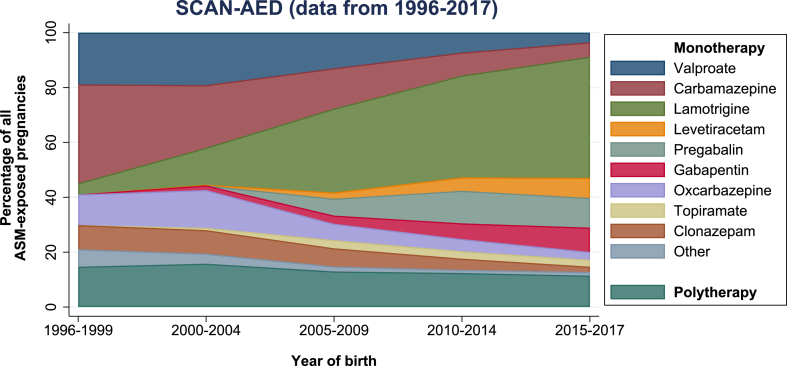
Proportion of antiseizure medication use in pregnancy in Denmark, Finland, Iceland, Norway, and Sweden by year of birth from 1996 to 2017. SCAN-AED: Nordic population-based cohort study including register data from Denmark, Finland, Iceland, Norway, and Sweden (www.scanaed.org). ASM: antiseizure medication.

We identified 4,494,918 children (males: 2,306,991 [51.3%]), including 27,070 (0.6%) children prenatally exposed to maternal ASMs. We identified 38,714 (0.9%) children born of mothers with epilepsy including 16,487 (0.4%) children prenatally exposed to ASMs (Fig. 2 and Figure S2). From 1996 to 2017, valproate, carbamazepine and lamotrigine were the ASMs most widely used in pregnancy, but with significant time trends; the use of lamotrigine in pregnancy increased, whereas the use of valproate and carbamazepine decreased with time. There was only a minor time trend in the use of ASMs in polytherapy (Fig. 2). Characteristics of children in the total population and children of mothers with epilepsy according to their prenatal exposure to ASMs are shown in Table 1.

**Table 1:** Characteristics of 4,494,918 children exposed and unexposed to antiseizure medication (ASM) in pregnancy, including 38,714 children of mothers with epilepsy.

	Overall population of children		Children of women with epilepsy	
	No ASM	Any ASM	No ASM	Any ASM
	N = 4,467,848	N = 27,070	N = 22,227	N = 16,487
**Country of birth, row percentage**^b^				
Denmark	1,233,649 (99.5)	6641 (0.5)	9393 (66.4)	4755 (33.6)
Finland	1,164,641 (99.3)	7939 (0.7)	1226 (20.8)	4668 (79.2)
Iceland	58,585 (99.1)	539 (0.9)	91 (33.8)	178 (66.2)
Norway	739,052 (99.4)	4464 (0.6)	6106 (67.4)	2953 (32.6)
Sweden	1,271,921 (99.4)	7487 (0.6)	5411 (57.9)	3933 (42.1)
**Year of birth, column percentage**				
1996–1999	404,341 (9)	1875 (7)	402 (2)	1053 (6)
2000–2004	579,178 (13)	2704 (10)	1458 (7)	2075 (13)
2005–2009	1,295,617 (29)	6837 (25)	6488 (29)	4794 (29)
2010–2014	1,405,415 (32)	9633 (36)	8555 (39)	5528 (34)
2015–2017	783,297 (18)	6021 (22)	5324 (24)	3037 (18)
**Sex of child**				
Female	2,174,733 (49)	13,194 (49)	10,830 (49)	8003 (49)
Male	2,293,115 (51)	13,876 (51)	11,397 (51)	8484 (52)
**Maternal age, years**				
<20	73,681 (2)	488 (2)	498 (2)	327 (2)
20–24	581,232 (13)	3833 (14)	3611 (16)	2252 (14)
25–29	1,399,551 (31)	8326 (31)	6950 (31)	5127 (31)
30–34	1,509,254 (34)	8563 (32)	6968 (31)	5470 (33)
35–39	741,959 (17)	4685 (17)	3487 (16)	2742 (17)
≥40	162,118 (4)	1175 (4)	713 (3)	569 (4)
Missing	53 (0)	0 (0)	0 (0)	0 (0)
**Maternal parity**				
0	1,922,962 (43)	12,646 (47)	9847 (44)	7670 (47)
1	1,601,282 (36)	8480 (31)	7716 (35)	5578 (34)
≥2	920,833 (21)	5764 (21)	4611 (21)	3143 (19)
Missing	22,771 (1)	180 (1)	53 (0)	96 (1)
**Hospital admittances in the year before pregnancy**				
0	3,789,990 (85)	20,339 (75)	17,138 (77)	12,644 (77)
1	559,049 (13)	4303 (16)	3563 (16)	2601 (16)
≥2	118,809 (3)	2428 (9)	1526 (7)	1242 (8)
**Maternal education**				
Compulsory	612,332 (14)	6569 (24)	5839 (26)	3411 (21)
Secondary/pre-university	2,076,296 (47)	13,038 (48)	9514 (43)	7923 (48)
Bachelor	979,439 (22)	4504 (17)	4233 (19)	3208 (20)
Master/PhD	614,555 (14)	2074 (8)	2060 (9)	1403 (9)
Missing^a^	185,226 (4)	885 (3)	581 (3)	542 (3)
**Smoking in early pregnancy**				
No	3,625,774 (81)	19,038 (70)	16,840 (76)	12,653 (77)
Yes	486,215 (11)	5788 (21)	3933 (18)	2596 (16)
Missing^a^	355,859 (8)	2244 (8)	1454 (7)	1238 (8)
**Mother married or cohabiting**				
No	330,741 (7)	3794 (14)	2524 (11)	1723 (11)
Yes	4,056,989 (91)	22,770 (84)	19,477 (88)	14,537 (88)
Missing	80,118 (2)	506 (2)	226 (1)	227 (1)
**Maternal psychiatric disorder**				
No	4,157,209 (93)	17,783 (66)	16,245 (73)	13,367 (81)
Yes	310,639 (7)	9287 (34)	5982 (27)	3120 (19)
**Use of psychotropic drugs in pregnancy**				
No	4,315,101 (97)	19,571 (72)	20,163 (91)	14,340 (87)
Yes	152,747 (3)	7499 (28)	2064 (9)	2147 (13)
**Median gestational age in days (IQR)**	280 (273–287)	278 (270–285)	279 (271–286)	279 (271–286)

IQR: interquartile range.

a Variables with more than 2% missing were imputed.

b The variation between countries in the proportion of mothers with epilepsy using ASM during pregnancy reflects differences in data availability. In Finland and Iceland, diagnostic information was available only for one year before pregnancy, meaning the group identified with epilepsy likely reflects a population with active epilepsy and hence a higher real proportion of ASM usage in pregnancy. In Denmark, Norway, and Sweden, diagnostic information was available for several years before pregnancy, meaning that the group identified with epilepsy likely reflects women with a life-time diagnosis of epilepsy and hence a lower proportion of ASM usage in pregnancy.

### Birth weight, low birth weight (<2500 g), and SGA children from the overall population prenatally exposed and unexposed to ASMs

The largest adjusted birthweight difference for any individual drug, relative to the unexposed children, was −170 g (range, −170 g–134 g) (Table 2 and Table S2). For most of the ASMs, prenatal exposure was not associated with low birth weight (<2500 g), however, we observed an increased risk after prenatal exposure to carbamazepine (aOR: 1.46 (95% CI: 1.25–1.71)), pregabalin (aOR: 1.23 (95% CI: 1.03–1.47)), topiramate (aOR: 1.51 (95% CI: 1.09–2.10)), and clobazam (aOR: 3.65 (95% CI: 1.52–8.76)). Similarly for most of the other ASMs, prenatal exposure was not associated with increased risk of being born SGA, but the risk of being born SGA increased after prenatal exposure to carbamazepine (aOR: 1.25 (95% CI: 1.12–1.40)), pregabalin (aOR: 1.16 (95% CI: 1.02–1.31)), oxcarbazepine (aOR: 1.48 (95% CI: 1.28–1.71)), clonazepam (aOR: 1.27 (1.10–1.48)), and topiramate (aOR: 1.48 (95% CI: 1.18–1.85)).

**Table 2:** Birth weight, low birth weight (<2500 g), and small for gestational age in 4,494,918 children exposed and unexposed to antiseizure medication (ASM) in monotherapy during pregnancy.

Exposure groups	Exposed	Number of ASM prescriptions	Birth weight (gram)		Low birth weight (<2500 gram)		Small for gestational age	
	n	Median (IQR)	Mean (SD)	Adjusted difference (95% CI)	N (%)	Adjusted OR (95% CI)	N (%)	Adjusted OR (95% CI)
No ASM	4,467,848	0	3536 (553)	0.00 (ref)	141,442 (3.2)	1.00 (ref)	446,267 (10.0)	1.00 (ref)
Any ASM	27,070	ND	3441 (600)	−26 (−33 to −18)	1440 (5.3)	1.14 (1.07–1.22)	3479 (12.9)	1.13 (1.08–1.18)
**Monotherapies**								
Lamotrigine	8756	4 (2–6)	3482 (594)	26 (14–38)	395 (4.5)	0.94 (0.84–1.05)	920 (10.5)	0.91 (0.85–0.99)
Carbamazepine	3424	3 (2–4)	3450 (623)	−58 (−77 to −38)	198 (5.8)	1.46 (1.25–1.71)	428 (12.5)	1.25 (1.12–1.40)
Valproate	2669	3 (2–4)	3499 (612)	30 (9–51)	120 (4.5)	1.00 (0.83–1.21)	325 (12.2)	1.07 (0.95–1.21)
Pregabalin	2214	2 (1–4)	3377 (582)	−58 (−81 to −35)	136 (6.1)	1.23 (1.03–1.47)	318 (14.4)	1.16 (1.02–1.31)
Oxcarbazepine	1591	3 (3–4)	3448 (577)	−39 (−67 to −12)	79 (5)	1.23 (0.97–1.55)	241 (15.1)	1.48 (1.28–1.71)
Clonazepam	1358	2 (1–4)	3357 (578)	−63 (−93 to −34)	86 (6)	1.05 (0.84–1.33)	221 (16.3)	1.27 (1.10–1.48)
Gabapentin	1336	1 (1–3)	3392 (592)	−88 (−117 to −59)	76 (6)	1.25 (0.98–1.59)	168 (12.6)	1.13 (0.96–1.34)
Levetiracetam	1077	4 (3–5)	3451 (577)	−29 (−62 to 4)	50 (5)	1.12 (0.84–1.51)	125 (11.6)	1.08 (0.89–1.32)
Topiramate	638	2 (1–4)	3422 (611)	−66 (−108 to −24)	40 (6)	1.51 (1.09–2.10)	96 (15)	1.48 (1.18–1.85)
Phenobarbital	183	1 (1–2)	3343 (599)	−144 (−223 to −65)	14 (8)	1.52 (0.86–2.70)	29 (16)	1.46 (0.97–2.20)
Acetazolamide	127	1 (1–2)	3427 (511)	−43 (−137 to 50)	<5	NA	15 (12)	1.00 (0.58–1.73)
Phenytoin	81	3 (2–4)	3598 (654)	76 (−44 to 195)	6 (7)	1.94 (0.83–4.51)	7 (9)	0.79 (0.36–1.73)
Clobazam	44	NA	3379 (874)	−126 (−285 to 33)	6 (14)	3.65 (1.52–8.76)	5 (11)	1.14 (0.44–2.92)
Primidone	34	NA	3395 (530)	−120 (−301 to 61)	<5	NA	6 (18)	1.84 (0.74–4.55)
Zonizamide	19	NA	3351 (538)	−90 (−332 to 152)	<5	NA	<5	NA
Vigabatrin	17	NA	3518 (471)	46 (−211 to 302)	<5	NA	<5	NA
Ethosuximide	11	NA	3285 (731)	−170 (−488 to 148)	<5	NA	<5	NA
Lacosamide	9	NA	3567 (342)	134 (−239 to 506)	<5	NA	<5	NA

NA = Not analyzed due to low number.

ND = not analyzed.

IQR: interquartile range, all values of medians and IQRs are shared by at least 5 individuals.

Adjustment: Country of birth, year of birth, sex of child, maternal age, parity, cohabitation, pre-pregnancy hospital admittances, maternal education, smoking in pregnancy, maternal psychiatric disorders, maternal epilepsy, and use of psychotropic drugs in pregnancy.

Using robust standard errors to account for siblings only had minor impact on the confidence intervals.

### Birth weight, low birth weight (<2500 g), and SGA in children of mothers with epilepsy prenatally exposed and unexposed to ASMs

**Fig. 3:**
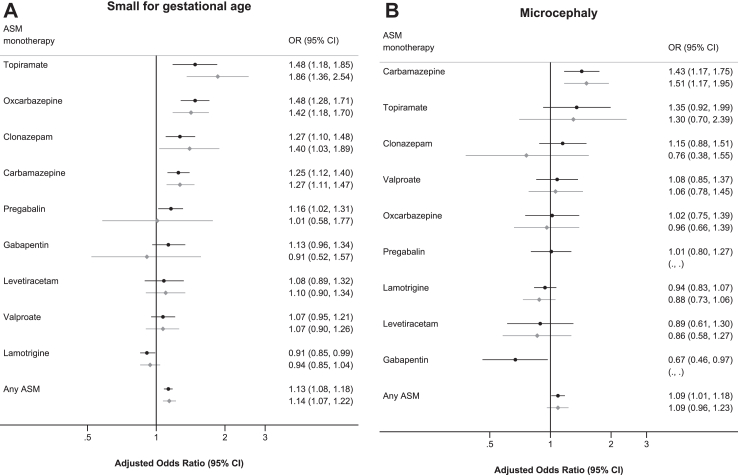
**a**) Risk of being born small for gestational age associated with monotherapy exposure to antiseizure medication (ASM) in pregnancy in all 4,494,918 children (black) and in the subset of 38,714 children of mothers with epilepsy (grey). **b)** Risk of microcephaly associated with monotherapy exposure to antiseizure medication (ASM) in pregnancy in all 4,494,918 children (black) and in the subset of 38,714 children of mothers with epilepsy (grey). ASM: Antiseizure medication; aOR: adjusted Odds Ratio. The figure shows the risk of small for gestational age and risk of microcephaly associated with prenatal exposure to antiseizure medication (ASM) for ASMs with more than 500 exposed children. Adjusted for country of birth, year of birth, sex of child, maternal age, parity, cohabitation, pre-pregnancy hospital admittances, maternal education, smoking in early pregnancy, maternal psychiatric disorders, and use of psychotropic drugs in pregnancy. Estimates in the full population are further adjusted for maternal epilepsy. There were too few children of mothers with epilepsy exposed to gabapentin and pregabalin to allow estimation of the risk of microcephaly.

In children of women with epilepsy, the largest adjusted birthweight difference for any individual drug, relative to the unexposed children, was 272 g (range, −183 g to 272 g), compared to unexposed children (Table 3). Again, for most ASM, prenatal exposure was not associated with low birth weight, but prenatal exposure to carbamazepine (aOR: 1.43 (95% CI: 1.16–1.75)) and topiramate (aOR: 1.66 (95% CI: 1.05–2.61)) was associated with increased risk of low birth weight (<2500 g). Also, in children of women with epilepsy, prenatal exposure to most ASMs was not associated with being born SGA, but prenatal exposure to carbamazepine (aOR: 1.27 (95% CI: 1.11–1.47)), oxcarbazepine (aOR: 1.42 (95% CI: 1.18–1.70)), and topiramate (aOR: 1.86 (95% CI: 1.36–2.54)) was associated with increased risks of being born SGA (Fig. 3a).

**Table 3:** Birth weight, low birth weight (<2500 g), and small for gestational age (SGA) in 38,714 children of mothers with epilepsy exposed and unexposed to antiseizure medication (ASM) in monotherapy during pregnancy.

Exposure groups	*Exposed*	Birth weight (gram)		Low birth weight (<2500 gram)		Small for gestational age	
	n	Mean (s.d.)	Adjusted mean difference (95% CI)	N (%)	Adjusted OR (95% CI)	N (%)	Adjusted OR (95% CI)
No ASM	22,227	3472 (583)	0.00 (ref)	1006 (4.5)	1.00 (ref)	2416 (10.9)	1.00 (ref)
Any ASM	16,487	3457 (606)	−15 (−28 to −2)	859 (5.2)	1.15 (1.04–1.27)	2118 (12.8)	1.14 (1.07–1.22)
**Monotherapies**							
Lamotrigine	5299	3499 (596)	27 (10–45)	230 (4.3)	0.97 (0.83–1.13)	546 (10.3)	0.94 (0.85–1.04)
Carbamazepine	2669	3449 (625)	−59 (−85 to −33)	153 (5.7)	1.43 (1.16–1.75)	341 (12.8)	1.27 (1.11–1.47)
Valproate	1953	3523 (620)	39 (9–69)	92 (5)	1.10 (0.85–1.41)	229 (11.7)	1.07 (0.90–1.26)
Pregabalin	120	3330 (558)	−35 (−139 to 69)	8 (7)	0.92 (0.44–1.92)	15 (13)	1.01 (0.58–1.77)
Oxcarbazepine	1462	3452 (581)	−33 (−68 to 3)	71 (5)	1.15 (0.86–1.54)	222 (15.2)	1.42 (1.18–1.70)
Clonazepam	339	3327 (610)	−79 (−141 to −16)	27 (8)	1.29 (0.85–1.95)	57 (17)	1.40 (1.03–1.89)
Gabapentin	138	3384 (553)	−46 (−141 to 49)	8 (6)	1.04 (0.50–2.14)	15 (11)	0.91 (0.52–1.57)
Levetiracetam	1063	3457 (573)	−28 (−63 to 8)	50^a^ (−)	1.08 (0.79–1.47)	125^a^ (−)	1.10 (0.90–1.34)
Topiramate	290	3369 (630)	−104 (−171 to −38)	21 (7)	1.66 (1.05–2.61)	53 (18)	1.86 (1.36–2.54)
Phenobarbital	47	3356 (554)	−129 (−293 to 34)	<5	NA	7 (15)	1.51 (0.67–3.43)
Acetazolamide	9	3260 (621)	−106 (−477 to 265)	<5	NA	<5	NA
Phenytoin	64	3541 (657)	54 (−88 to 197)	6^a^ (−)	1.94 (0.76–4.95)	7 (11)	0.91 (0.41–2.04)
Clobazam	27	3350 (921)	−118 (−333 to 97)	<5	NA	<5	NA
Primidone	27	3372 (535)	−113 (−328 to 102)	0	NA	6 (22)	2.24 (0.87–5.76)
Zonisamide	19	3351 (538)	−102 (−358 to 154)	<5	NA	<5	NA
Vigabatrin	9	3646 (415)	272 (−101 to 644)	0	NA	0	NA
Ethosuximide	11	3285 (731)	−183 (−519 to 153)	<5	NA	<5	NA
Lacosamide	9	3567 (342)	119 (−274 to 513)	0	NA	<5	NA

NA = Not analyzed due to low numbers.

Adjustment: Country of birth, year of birth, sex of child, maternal age, parity, cohabitation, pre-pregnancy hospital admittances, maternal education, smoking in pregnancy, maternal psychiatric disorders, and use of psychotropic drugs in pregnancy.

a Number is copied from Table 2 for data privacy reasons, as differences are <5. However, analyses are based on actual numbers.

### SGA by 3rd percentile in children from the overall population and in children of mothers with epilepsy prenatally exposed and unexposed to ASM

The aORs of being born SGA by 3rd percentile (Table S3) were similar to aORs of being born SGA by 10th percentile after ASM exposure in pregnancy (Tables 2 and 3).

### Head circumference and microcephaly in children from the overall population prenatally exposed and unexposed to ASMs

Prenatal exposure to most ASMs was not associated with head circumference, but prenatal exposure to carbamazepine (−0.27 cm (95% CI: −0.33 to −0.20)), pregabalin (−0.09 cm (95% CI: −0.16 to −0.02)), clonazepam (−0.15 cm (95% CI: −0.25 to −0.06)), gabapentin (−0.10 cm (95% CI: −0.19 to −0.01)), and phenobarbital (−0.45 cm (95% CI: −0.69 to −0.20)) was associated with a smaller head circumference, whereas prenatal exposure to vigabatrin (0.82 cm (95% CI: 0.03–1.62)) was associated with a larger head circumference (Table 4 and Table S4). Among the individual ASMs in monotherapy, only carbamazepine was associated with increased risk of microcephaly (aOR: 1.43 (95% CI: 1.17–1.75)).

**Table 4:** Head circumference and microcephaly in 4,494,918 children exposed and unexposed to antiseizure medication (ASM) in monotherapy during pregnancy.

Exposure groups	Exposed	Head circumference (centimeters)		Microcephaly	
	n	Mean (SD)	Adjusted difference (95% CI)	N^a^ (%)	Adjusted OR (95% CI)
No ASM	4,467,848	35.0 (1.8)	0.00 (ref)	134,023 (3.0)	1.00 (ref)
Any ASM	27,070	34.8 (1.9)	−0.06 (−0.08 to −0.04)	992 (3.7)	1.09 (1.01–1.18)
**Monotherapies**					
Lamotrigine	8756	34.9 (1.9)	0.06 (0.03 to 0.10)	274 (3.1)	0.94 (0.83–1.07)
Carbamazepine	3424	34.7 (2.1)	−0.27 (−0.33 to −0.20)	135 (3.9)	1.43 (1.17–1.75)
Valproate	2669	34.9 (1.9)	0.03 (−0.04 to 0.09)	97 (3.6)	1.08 (0.85–1.37)
Pregabalin	2214	34.6 (1.8)	−0.09 (−0.16 to −0.02)	83 (3.8)	1.01 (0.80–1.27)
Oxcarbazepine	1591	34.9 (1.8)	−0.03 (−0.12 to 0.06)	52 (3.2)	1.02 (0.75–1.39)
Clonazepam	1358	34.6 (1.9)	−0.15 (−0.25 to −0.06)	61 (4.5)	1.15 (0.88–1.51)
Gabapentin	1336	34.8 (1.8)	−0.10 (−0.19 to −0.01)	32 (2.4)	0.67 (0.46–0.97)
Levetiracetam	1077	34.9 (1.8)	0.02 (−0.08 to 0.12)	32 (2.9)	0.89 (0.61–1.30)
Topiramate	638	34.8 (1.8)	−0.05 (−0.18 to 0.08)	28 (4.3)	1.35 (0.92–1.99)
Phenobarbital	183	34.5 (1.8)	−0.45 (−0.69 to −0.20)	11 (6.3)	1.82 (0.96–3.47)
Acetazolamide	127	35.0 (1.5)	0.15 (−0.14 to 0.44)	0	NA
Phenytoin	81	35.2 (2.1)	0.12 (−0.29 to 0.54)	<5	NA
Clobazam	44	34.8 (2.5)	−0.19 (−0.69 to 0.31)	0	NA
Primidone	34	34.6 (1.5)	−0.45 (−1.02 to 0.11)	<5	NA
Zonizamide	19	34.2 (1.8)	−0.61 (−1.35 to 0.13)	<5	NA
Vigabatrin	17	35.7 (1.7)	0.82 (0.03 to 1.62)	<5	NA
Ethosuximide	11	34.4 (2.5)	−0.38 (−1.36 to 0.59)	0	NA
Lacosamide	9	35.4 (1.4)	0.79 (−0.35 to 1.93)	<5	NA

NA = Not analyzed due to low numbers.

Adjustment: Country of birth, year of birth, sex of child, maternal age, parity, cohabitation, pre-pregnancy hospital admittances, maternal education, smoking in pregnancy, maternal psychiatric disorders, maternal epilepsy, and use of psychotropic drugs in pregnancy. Using robust standard errors to account for siblings only had minor impact on the confidence intervals.

a Rounded average of 20 imputed N's.

### Head circumference and microcephaly in children of mothers with epilepsy prenatally exposed and unexposed to ASMs

In children of women with epilepsy, prenatal exposure to most ASMs was not associated with head circumference, but prenatal exposure to carbamazepine (−0.32 cm (95% CI: −0.40 to −0.23)) and phenobarbital (−0.59 cm (95% CI: −1.90 to −0.08)) was associated with smaller head circumference, whereas we observed a larger head circumference after prenatal exposure to lamotrigine (0.07 cm (95% CI: 0.02–0.13)) and vigabatrin (1.28 cm (95% CI: 0.12–2.43)) (Table 5). Among children of women with epilepsy, prenatal exposure to most ASMs was not associated with microcephaly, but prenatal exposure to carbamazepine was associated with increased risk of microcephaly (aOR: 1.51 (95% CI: 1.17–1.95)) (Fig. 3b).

**Table 5:** Head circumference and microcephaly in 38,714 children of mothers with epilepsy exposed and unexposed to antiseizure medication (ASM) in monotherapy during pregnancy.

Exposure groups	Exposed	Head circumference, cm		Microcephaly	
	n	Mean (s.d.)	Adjusted mean difference (cm) (95% CI)	N^a^ (%)	Adjusted OR (95% CI)
No ASM	22,227	34.9 (1.8)	0.00 (ref)	699 (3.1)	1.00 (ref)
Any ASM	16,487	34.8 (1.9)	−0.06 (−0.09 to −0.02)	588 (3.6)	1.09 (0.96–1.23)
**Monotherapies**					
Lamotrigine	5299	34.9 (1.9)	0.07 (0.02 to 0.13)	151 (2.8)	0.88 (0.73–1.06)
Carbamazepine	2669	34.7 (2.0)	−0.32 (−0.40 to −0.23)	105 (3.9)	1.51 (1.17–1.95)
Valproate	1953	34.9 (1.9)	0.04 (−0.06 to 0.13)	67 (3.4)	1.06 (0.78–1.45)
Pregabalin	120	34.5 (1.7)	−0.12 (−0.44 to 0.20)	<5	NA
Oxcarbazepine	1462	34.9 (1.8)	0.02 (−0.10 to 0.13)	48 (3.3)	0.96 (0.66–1.39)
Clonazepam	339	34.6 (1.9)	−0.15 (−0.34 to 0.05)	9 (2.7)	0.76 (0.38–1.55)
Gabapentin	138	34.7 (1.8)	−0.14 (−0.44 to 0.16)	<5	NA
Levetiracetam	1063	34.9 (1.8)	0.02 (−0.09 to 0.13)	31 (2.9)	0.86 (0.58–1.27)
Topiramate	290	34.8 (1.9)	−0.10 (−0.31 to 0.11)	11 (3.9)	1.30 (0.70–2.39)
Phenobarbital	47	34.4 (1.5)	−0.59 (−1.09 to −0.08)	<5	NA
Acetazolamide	9	34.6 (1.9)	−0.11 (−1.26 to 1.04)	0	NA
Phenytoin	64	35.1 (2.2)	0.04 (−0.43 to 0.51)	<5	NA
Clobazam	27	34.7 (2.8)	−0.20 (−0.87 to 0.46)	0	NA
Primidone	27	34.5 (1.5)	−0.48 (−1.16 to 0.20)	<5	NA
Zonisamide	19	34.2 (1.8)	−0.64 (−1.43 to 0.15)	<5	NA
Vigabatrin	9	36.0 (1.2)	1.28 (0.12 to 2.43)	0	NA
Ethosuximide	11	34.4 (2.5)	−0.43 (−1.47 to 0.61)	0	NA
Lacosamide	9	35.4 (1.4)	0.77 (−0.45 to 1.99)	<5	NA

NA = Not analyzed due to low numbers.

Adjustment: Country of birth, year of birth, sex of child, maternal age, parity, cohabitation, pre-pregnancy hospital admittances, maternal education, smoking in pregnancy, maternal psychiatric disorders, and use of psychotropic drugs in pregnancy.

a Rounded average of 20 imputed Ns.

### Dose response analyses in children from the overall population prenatally exposed and unexposed to ASMs

Prenatal exposure to lamotrigine, pregabalin and gabapentin was not associated with SGA in neither high nor low dose. Prenatal exposure to high-dose carbamazepine (aOR: 1.32 (95% CI: 1.15–1.51)), high-dose oxcarbazepine (aOR: 1.64 (95% CI: 1.41–1.92)), high-dose clonazepam (aOR: 2.66 (95% CI: 1.86–3.81)), low-dose topiramate (aOR: 1.30 (95% CI: 1.00–1.68)), and high-dose topiramate (aOR: 2.34 (95% CI: 1.50–3.65)) was associated with increased risks of being SGA (Table S5). Prenatal exposure to low dose (aOR: 1.51 (95% CI: 1.04–2.17)), but not high dose (aOR: 0.97 (95% CI: 0.77–1.22)) levetiracetam was associated with increased risk of being SGA. Only prenatal exposure to high dose carbamazepine was associated risk of microcephaly (aOR: 1.60 (95% CI: 1.25–2.06)) (Table S5).

### Risk of fetal growth restriction in children of women without epilepsy prenatally exposed and unexposed to ASMs

In 4,456,204 children of women without epilepsy, prenatal exposure to pregabalin (aOR: 1.17 (95% CI: 1.03–1.32)) and clonazepam (aOR: 1.22 (95% CI: 1.02–1.45)) was associated with being SGA (Table S6), but when assessing microcephaly, we found no statistically significant associations with prenatal ASM exposures (Table S7).

### SGA and microcephaly for children prenatally exposed and unexposed to ASMs in mono- and polytherapy combined

In analyses including children exposed to ASMs in mono- and polytherapy (i.e., not restricting to monotherapy), exposure to carbamazepine (aOR: 1.35 (95% CI: 1.22–1.49)), valproate (aOR: 1.12 (95% CI: 1.01–1.25)), pregabalin (aOR: 1.17 (95% CI: 1.05–1.32)), oxcarbazepine (aOR: 1.80 (95% CI: 1.60–2.03)), clonazepam (aOR: 1.30 (95% CI: 1.15–1.47)), gabapentin (aOR: 1.19 (95% CI: 1.02–1.38)), levetiracetam 1.30 (95% CI: 1.14–1.49)), topiramate (aOR: 1.70 (95% CI: 1.44–2.01)), and clobazam (aOR: 1.45 (95% CI: 1.09–1.94) was associated with SGA (Table S8). Additional analyses of other drugs used in mono- and polytherapy are shown in Tables S9–S11.

### SGA and microcephaly for children of mothers with “active” epilepsy prenatally exposed and unexposed to ASMs

In analyses restricting to children of mothers with “active” epilepsy, prenatal exposure to carbamazepine (aOR: 1.22 (1.05–1.42)), oxcarbazepine (aOR: 1.30 (1.05–1.59)), and topiramate (aOR: 1.83 (1.31–2.55)) was associated with being born small for gestational age; carbamazepine, with microcephaly (aOR: 1.38 (1.05–1.82)) (Table S12).

### Complete case analyses of SGA and microcephaly for children prenatally exposed and unexposed to ASMs

In complete analyses, prenatal exposure to carbamazepine (aOR: 1.27 (1.10–1.45)), oxcarbazepine (aOR: 1.37 (1.15–1.64)), clonazepam (aOR: 1.26 (1.05–1.50)) and topiramate (aOR: 1.55 (1.23–1.95)) was associated with being born small for gestational age; carbamazepine, with microcephaly (aOR: 1.58 (1.26–1.97)) (Table S13).

### SGA and microcephaly for children prenatally exposed and unexposed to ASMs after restricting the exposure period to the period from the first day of the last menstrual period to birth

In analyses with a restricted exposure period, prenatal exposure to carbamazepine (aOR: 1.25 (1.12–1.40)), pregabalin (aOR: 1.22 (1.06–1.40), oxcarbazepine 1.49 (1.29–1.72)), clonazepam (aOR: 1.32 (1.13–1.54)) and topiramate (aOR: 1.69 (1.33–2.16)) was associated with being born small for gestational age; and carbamazepine (aOR: 1.58 (1.26–1.97)) and phenobarbital (aOR: 1.90 (1.00–3.63)) with microcephaly (Tables S14).

### Head circumference and microcephaly in children from the overall population prenatally exposed and unexposed to ASMs after excluding 195,543 children with congenital malformations

After excluding children with congenital malformation (Table S15), the association of prenatal exposure to ASMs with head circumference and microcephaly was almost identical to the estimates including the entire population (Tables 4 and 5). Among the individual ASMs in monotherapy, only carbamazepine was associated with increased risk of microcephaly (aOR: 1.46 (95% CI: 1.19–1.79)).

### SGA and microcephaly for children from the overall population and in children of mothers with epilepsy exposed and unexposed to carbamazepine and lamotrigine in monotherapy during pregnancy

In the overall population, prenatal exposure to carbamazepine compared to lamotrigine was associated with increased risk of SGA (aOR = 1.37 (1.17–1.60)) and microcephaly (aOR = 1.78 (1.36–2.32)) (Table S16). In children of mothers with epilepsy, prenatal exposure to carbamazepine compared to lamotrigine was associated with increased risk of SGA (aOR = 1.39 (1.16–1.65)) and microcephaly (aOR = 1.70 (1.25–2.31)).

### SGA and microcephaly for children from the overall population born of mothers with information on BMI exposed and unexposed to ASMs

In analyses restricted to children born of mothers with information of BMI, prenatal exposure to carbamazepine (aOR: 1.32 (1.14–1.52)), pregabalin (aOR: 1.19 (1.05–1.35)), oxcarbazepine (aOR: 1.28 (1.06–1.54)), clonazepam (aOR: 1.33 (1.10–1.60)) and topiramate (aOR: 1.40 (1.10–1.80)) was associated with being born small for gestational age; and carbamazepine (aOR: 1.66 (1.31–2.11)) and phenobarbital (aOR: 2.97 (1.25–7.03)) with microcephaly (Tables S17). After additionally adjusting for maternal BMI, the risk estimates were almost identical to the estimates without adjusting for maternal BMI (Tables S17).

## Discussion

In this large population-based study of fetal growth, we found that prenatal monotherapy exposure to carbamazepine, oxcarbazepine, clonazepam, and topiramate was consistently associated with risk of being born with a low birth weight and being born SGA in both the overall population and in children of women with epilepsy. It was possible to adjust for potential confounders including psychiatric co-morbidity and use of psychotropic drugs in pregnancy. The findings remained in sensitivity analyses, i.e., when further restricting to children born of women with “active” epilepsy, in complete case analyses, when restricting the exposure period to the period from the first day of the last menstrual period to birth, and after excluding children with congenital malformations. Carbamazepine was the only ASM consistently associated with increased risk of microcephaly. Prenatal exposure to phenobarbital was associated with low birth weight and small head circumference, but aOR of SGA and microcephaly was only of borderline significance, possibly due to the low number of exposed children. These findings raise concern of the short- and long-term consequences of prenatal ASM exposure and risk of fetal growth restriction.^1^^,^^2^ Infants born SGA are at increased risk of perinatal morbidity and metabolic alterations in later life, which may contribute to burden from obesity, type 2 diabetes, hypertension, and cardiovascular disease.^23^

Prenatal carbamazepine exposure has previously been associated with risk of being born SGA^3^, ^4^, ^5^, ^6^, ^7^^,^^10^ and with microcephaly.^6^^,^^10^ including a study from Denmark with a subset of the data included in the current study.^3^ In addition, studies of prenatal carbamazepine exposure have shown evidence of fetal growth restriction in rodents.^12^^,^^13^ Still, the evidence for an association of fetal growth restriction with prenatal carbamazepine exposure was considered inconclusive before this study,^4^ and the risk is not mentioned in the prescribing information for carbamazepine.^24^ In two population-based studies based on subsets of data from the current study, prenatal oxcarbazepine exposure was associated with risk of being born SGA,^5^^,^^25^ although fetal growth restriction was not found in a meta-analysis that did not include these two studies.^26^ Oxcarbazepine exposure in rats during the latter part of gestation and throughout the lactation period was associated with reduced body weight.^14^ Potential human risk of fetal growth restriction is not mentioned in the prescribing information for oxcarbazepine.^14^ SGA is a statistical definition of a deviation of size measurement, and although it is therefore an indicator, it is not identical to fetal growth restriction.^19^ A recent attempt to identify fetal growth restriction has suggested that birth weight less than the 3rd percentile may better identify growth restriction in the newborn.^19^ However, using birth weight less than the 3rd percentile as outcome we also found that prenatal exposure to carbamazepine and oxcarbazepine was associated with growth restriction in the newborn. For clonazepam, very little human data is available,^4^^,^^8^ but the prescribing information mentions reduction in embryofetal growth in rabbits.^15^ The risk of fetal growth restriction is better established for topiramate, thus, the risk of being born SGA has been shown in various human studies, including previous studies based on subsets of data from the current study,^5^^,^^27^ and in multiple animal species.^16^ Accordingly, the prescribing information for topiramate provides information about these risks associated with prenatal exposure.^16^ Reassuringly, lamotrigine was not associated with fetal growth restriction, thus underpinning the evidence of the safety of this drug in pregnancy.^4^ In dose response analyses—taking cumulative ASM exposure in pregnancy into account–prenatal exposure to high dose ASM was, compared to low dose ASM, generally associated with higher risk of indicators for reduced fetal growth. However, prenatal exposure to low dose, but not high dose, of levetiracetam was associated with risk of being born SGA. The absence of a clear dose–response relationship does not support that prenatal exposure to levetiracetam is causally associated with an increased risk of being born SGA. In general, it is difficult to estimate daily dose of ASM from prescription fill data, and we did not have data from therapeutic drug monitoring. Prenatal exposure to pregabalin was associated with SGA in children from the overall population. However, the number of exposed children of mothers with epilepsy was too low to assess the risk in this population. Pregabalin is mostly used for pain and anxiety, and recently was re-classified as an analgesic (https://www.who.int/tools/atc-ddd-toolkit/about-ddd), thus it is not surprising that the number of mothers with epilepsy filling prescriptions for pregabaline in pregnancy is low. The present study assessed the risks of fetal growth restriction associated with prenatal exposure also to several new or less frequently used ASMs, such as phenobarbital, acetazolamide, phenytoin, clobazam, primidone, zonisamide, vigabatrin, ethosuximide and lacosamide. Hardly any human pregnancy safety data regarding fetal growth restriction has been available for these substances. However, even in our large study, the statistical power was insufficient to study most of these ASMs.

We used high-quality, unselected, nationwide data from five countries and were able to present the largest study to date of prenatal exposure to specific ASMs and the associated risk of fetal growth restriction. The study confirms and expands previous reports based on subsets of the data used for the present study which may constitute a limitation of this study.^5^^,^^25^^,^^28^ Even so, the sample size translated into more precise estimates of the risks associated with specific ASMs commonly used in pregnancy, and our findings were consistent across analyses and when restricting to offspring of women with epilepsy. We imputed missing information on education, smoking, and head circumference. The multiple imputation method assumes that information is missing at random, but even with multiple covariates included in the imputation model, we cannot exclude that there could be some deviation from the missing at random assumption, which cannot necessarily be easily corrected by adding more covariates. However, the estimates from analyses with imputed data (Tables 2 and 4) were very similar to the estimates from complete case analyses (Table S13), suggesting that this is unlikely to have caused substantial bias. For head circumference, measurements could possibly more often be missing when the child's head is malformed and either extremely large or extremely small. In this case, the imputation of head circumference could introduce bias and is a limitation to the study. However, in sensitivity analyses where we excluded children with congenital malformations, the association of prenatal exposure to ASMs with head circumference and microcephaly was almost identical to the estimates including the entire population, and we therefore do not think this is a major source of bias in this study. It is also possible that head circumference is missing in the children who experience birth complications due to a large head of the child, i.e., when there are acute complications during delivery (e.g., birth asphyxia) resulting in failure to measure head circumference at birth. If ASM exposure in pregnancy is associated with complications during delivery, this will result in underestimation of a potential risk of microcephaly associated with ASM exposure in pregnancy.

Observational studies are the only option to estimate effects of interventions during pregnancy, as pregnant women are usually excluded from randomized drug trials performed prior to approval. Attempts to emulate randomized trials may overcome some of the confounding issues in observational studies,^29^ however, this approach require that we are able to identify exposures initiated in the weeks following conception. This will only rarely be the case in pregnant women with epilepsy.

When assessing SGA, we used gestational age in weeks rather than in days. It is possible that there may be some misclassification, i.e., children born in the beginning of a gestational week (e.g., 39 + 0 or 39 + 1) who might be just below the 10th percentile when using whole weeks, could be just above the 10th percentile if we used days. However, sensitivity analysis using the cut-off for SGA at the 3rd percentile is so restrictive, that even with slight misclassification around the cut-off, these children are likely to be growth restricted—and the signal and thus conclusion in these analyses were the same as for the primary analyses using the 10th percentile as cut off for SGA. Nevertheless, in both analyses, our approach introduces potential for differential misclassification, which could potentially explain part of the reported associations if there were more planned labor inductions in women treated with ASM in the early part of a gestational week and this may constitute a limitation of this study. However, such bias would be expected to affect estimates of all ASMs, and since the associations with SGA only pertained to specific ASMs, we believe that this is unlikely to have had any major impact. We identified unlikely values of birth weight for live births and unlikely values of head circumference. There are multiple ways to identify implausible values of birth weight, gestational age and head circumference and there is no uniformly accepted consensus on how to do this. However, our approach may be less sensitive than more extensive approaches based on gestational age,^30^^,^^31^ If our approach is insufficient, it may introduce bias, but we assume that this potential bias is non-differential in relation to ASM exposure and therefore not a major limitation in this study. We assessed indicators of fetal growth restriction and found similar risk in offspring in the whole population and in the subset of children born of women with epilepsy suggesting that confounding by indication may not be a major issue in this study. This is supported by our analyses of prenatal exposure to carbamazepine, for which we used prenatal exposure to lamotrigine as an active comparator, which revealed similar findings and do not indicate significant bias from confounding by indication. Women with epilepsy who are pregnant are at increased risk of perinatal complication, which may also influence birth outcomes including birth weight. However, we accounted for these complications through adjustment for maternal epilepsy. Pregnancy complications that could be due to ASM exposure itself are mediators rather than confounders and should not be adjusted for. We used information on maternal filled prescriptions to measure prenatal ASM exposure. Therefore, we cannot be certain that the women consumed the dispensed medication in the period of interest. However, Nordic validation studies have shown that a high agreement exists between information from national registers of drugs prescribed for chronic diseases such as ASMs and the use of drugs self-reported to be used daily by pregnant women.^32^, ^33^, ^34^ Due to the large number of ASMs analyzed in this study (18 monotherapies and 27 mono/poly-therapies), we did not perform separate imputations for each ASM, but rather used the overall ASM variable (any use, yes/no) in the model, which may be a limitation of this study. However, we did evaluate the impact of using an ASM-specific indicator in the imputation model (instead of the overall binary ASM indicator) in analyses of carbamazepine monotherapy and SGA and microcephaly, and only found negligible differences (data not shown). Including only a binary variable of any ASMs in the imputation model is likely to result in regression towards the estimate of any ASM.

The available data did not include information on seizures in pregnancy,^35^ folic acid supplementation, and maternal nutrition status. Seizures during pregnancy may be considered a mediator of the potential effect of ASMs on fetal growth. However, as we did not have access to direct information on maternal seizure control during pregnancy, we are not able to address this further e.g., in mediation analyses. We did not include information on use of folic acid in this study as this information was not available from Finland. Folic acid supplementation in pregnancy has been associated with reduced risk of being born SGA.^36^ However, use of folic acid supplementation is much more frequent in mothers with epilepsy than in mothers without epilepsy,^37^ i.e., use of ASMs increases the probability of being prescribed folic acid supplementation, and not the other way around. Folic acid supplementation may thus be considered a mediator rather than a confounder and accordingly should not be adjusted for. We accounted for a range of potential confounders. Nonetheless, unmeasured confounding may still influence our estimates, and we did not have information on several potential confounders including maternal nutrition status, substance abuse including alcohol, and ethnicity and race. Maternal BMI may be a confounder in the study, but information on maternal BMI was missing from 29% of the population and data on maternal BMI was not available from Iceland in this study.^37^ Ideally, information from these variables, potentially resulting in unmeasured confounding, could provide more estimates of the association of prenatal exposure to ASM and indicators of fetal growth. Maternal BMI may influence the choice of ASM and influence fetal growth, but in analyses restricted to children with information on maternal BMI, the risk estimates of SGA and microcephaly were almost identical to the estimates without adjusting for maternal BMI. This suggests that the lack of adjustment for maternal BMI in the main analyses was not an important cause of bias in this study.

The study may be subject to selection bias including live birth bias. Prenatal exposure to ASMs have been associated with abortions and still birth,^38^ which could attenuate the “true” association between prenatal exposure to ASMs and indicators of fetal growth.

In the present study, prenatal exposure to several ASMs was associated with indicators of fetal growth restriction, and the affected children may potentially suffer from long-term consequences.^1^^,^^2^ Data on the long-term consequences of prenatal exposure to specific ASMs is limited. However, prenatal exposure to ASM was linked to adverse and delayed neurodevelopment in exposed children.^17^ The extent to which these risks can be attributed to fetal growth restriction remains uncertain. Valproate, the ASM for which evidence on prenatal exposure and risk of neurodevelopmental disorders is most robust,^17^ was not associated with fetal growth restriction in the present study. Additional mechanisms most probably contribute to the adverse neurodevelopment associated with ASM use by the mother during pregnancy.

In conclusion, this is the largest study to assess the association between prenatal exposure to ASMs and indicators of fetal growth. The study provides new insight and confirms concerns raised in previous reports from smaller studies. For the ASMs most often used in pregnant women, lamotrigine seems safe with regards to fetal growth, whereas the study raises concern for carbamazepine, oxcarbazepine, clonazepam and topiramate. The study informs patients and doctors, and the results may be used in pregnancy consultation when discussing the complex issues of maternal health in pregnancy and potential risk of ASMs associated with fetal growth. The findings should be interpreted in the light of other potential adverse outcomes associated with prenatal exposure to ASMs including risk of adverse neurodevelopment and congenital malformations.

## Contributors

Marte-Helene Bjørk, Jakob Christensen, Helga Zoega, and Torbjörn Tomson obtained funding for the study, Marte-Helene Bjørk, Mikka Gissler, Helga Zoega, Torbjörn Tomson and Jakob Christensen provided administrative, technical, or material support for the study, Jannicke Igland, Julie Werenberg Dreier and Jakob Christensen developed the study concept and design, all authors participated in the acquisition, analysis, and interpretation of data, Jannicke Igland and Julie Werenberg Dreier had full access to all the data in the study and take responsibility for the integrity of the data and the accuracy of the data analysis, Jakob Christensen and Julie Werenberg Dreier drafted the first version of the manuscript and supervised the study, all authors participated in critical revision of the manuscript for important intellectual content.

## Data sharing statement

Data were based on Nordic national registers and individual level data cannot be shared due to national regulations. However, summary statistics in addition to the results provided in the results section and supplementary material, may be provided upon request. Original data are available upon application to the relevant authorities.

## Declaration of interests

Jakob Christensen reports funding from the Danish Epilepsy Association, the Central Denmark Region, the Lundbeck Foundation (R400-2022-1205), and the Novo Nordisk Foundation (NNF16OC0019126 and NNF22OC0075033); Speaker honoraria from Eisai AB and UCB Nordic; Scientific advisory board honoraria from Eisai AB and UCB Nordic.

Marte-Helene Bjørk reports funding from the NordForsk Nordic Program on Health and Welfare (project no. 83796) and the Norwegian Research Counsil; consulting fees from Novartis Norway, Eisai (advisory board), Jazz Pharmaceuticals, Lundbeck and Angelini Pharma; Speaker honoraria from Eisai, AbbVie, Teva and Lilly.

Silje Alvestad reports funding from the NordForsk Nordic Program on Health and Welfare (project no. 83796) and speaker honoraria from Eisai AB.

Mika Gissler reports funding from the Research Council of Norway (International Pregnancy Drug Safety Studies project no. 273366), and the European Union: the Innovative Medicines Initiative (project no: 821520).

Jannicke Igland reports support from Sanofi and Novartis to conduct post-marketing drug safety research not related to the submitted work.

Maarit K. Leinonen reports funding from the Innovative Medicines Initiative (project no: 821520), the Finnish Medicines Agency (Fimea) and the Research Council of Norway (International Pregnancy Drug Safety Studies project no. 273366).

Nils Erik Gilhus reports honoraria from UCB, Janssen, Argenx, Merck, Alexion, Immunovant, Denka, Roche, Dianthus, and participation on an advisory board from UCB, Janssen, Argenx, Merck, Alexion, Immunovant, and Dianthus; all unrelated to the present study.

Yuelian Sun reports funding from the Independent Research Fund Denmark (9039-00296B).

Helga Zoega was supported by a UNSW Scientia Program Award (no number attached).

Torbjörn Tomson reports funding from Accord, Glenmark, GSK, UCB, Eisai, Ecu Pharma, Bial, Teva, Sanofi, SF Group, GW Pharma, Zentiva, and Angelini as donations to the EURAP pregnancy registry; Speaker honoraria from Eisai, Angelini, GSK and UCB.

Julie Werenberg Dreier reports funding from the NordForsk Nordic Program on Health and Welfare (project no. 83796) and the Independent Research Fund Denmark (1133-00026B and 316-00134A).

## Appendix A Supplementary data

Supplementary Material

Translated Abstracts
