# Supplementary material for: Prenatal exposure to antiseizure medications and fetal growth: a population-based cohort study from the Nordic countries
Source: Lancet Reg Health Eur. 2024 Feb 8;38:100849. doi: 10.1016/j.lanepe.2024.100849 (PMC10928302; doi:10.1016/j.lanepe.2024.100849)
Supplement: Supplementary Material [file mmc1.docx]

**Supplementary Appendix**

[**List of investigators** 3](#_Toc155547673)

[**E-methods** 4](#_Toc155547674)

[**Figure S1.** Proportion and number of users of anti-seizure medication in pregnancy in Denmark, Finland, Norway, and Sweden by year of birth. 5](#_Toc155547675)

[**Table S1a** Data sources from five Nordic countries (Denmark, Finland, Iceland, Norway, and Sweden) 6](#_Toc155547676)

[**Table S1b** Variables from five Nordic countries (Denmark, Finland, Iceland, Norway, and Sweden) 7](#_Toc155547677)

[**Table S1c:** Ethical and/or data protection authorities that have approved the project in all countries 9](#_Toc155547678)

[**Table S2.** Unadjusted and adjusted estimates of birth weight, low birth weight (<2,500 grams), and small for gestational age in 4,494,918 children exposed and unexposed to antiseizure medication (ASM) in monotherapy during pregnancy 10](#_Toc155547679)

[**Table S3.** Small for gestational age (SGA) by 3rd percentile in 4,494,918 children from the overall population and in the subset of 38,714 children of mothers with epilepsy prenatally exposed and unexposed to antiseizure medication (ASM) in monotherapy during pregnancy 12](#_Toc155547680)

[**Table S4.** Unadjusted and adjusted estimates of head circumference and microcephaly in 4,494,918 children exposed and unexposed to antiseizure medication (ASM) in monotherapy during pregnancy 14](#_Toc155547681)

[**Table S5.** Small for gestational age (SGA) and microcephaly in 4,494,918 children exposed and unexposed to high- and low-dose antiseizure medication (ASM) in monotherapy during pregnancy 16](#_Toc155547682)

[**Tables S6 and S7** Risk of restricted intrauterine growth in 4,456,204 children of women without epilepsy prenatally exposed and unexposed to ASMs. 18](#_Toc155547683)

[**Table S6.** Birth weight, low birth weight (<2,500 g), and small for gestational age (SGA) in 4,456,204 children of **mothers without epilepsy** exposed and unexposed to antiseizure medication (ASM) in monotherapy during pregnancy 19](#_Toc155547684)

[**Table S7.** Head circumference and microcephaly in 4,456,204 children of **mothers without epilepsy** exposed and unexposed to antiseizure medication (ASM) in monotherapy during pregnancy 20](#_Toc155547685)

[**Tables S8-S11** Risk of restricted intrauterine growth in children after prenatal exposure to ASMs in mono- and polytherapy combined. 22](#_Toc155547686)

[**Table S8**. Birth weight, low birth weight (<2,500 g), and small for gestational age (SGA) in 4,494,918 children exposed and unexposed to antiseizure medication (ASM) in **mono- and polytherapy** during pregnancy 23](#_Toc155547687)

[**Table S9.** Birth weight, low birth weight (<2,500 g), and small for gestational age (SGA) in 38,714 children of **mothers with epilepsy** exposed and unexposed to antiseizure medication (ASM) in **mono- and polytherapy** during pregnancy 25](#_Toc155547688)

[**Table S10.** Head circumference and microcephaly in 4,494,918 children exposed and unexposed to antiseizure medication (ASM) in **mono- and polytherapy** during pregnancy 27](#_Toc155547689)

[**Table S11.** Head circumference and microcephaly in 38,714 children of **mothers with epilepsy** exposed and unexposed to antiseizure medication (ASM) in **mono- and polytherapy** during pregnancy 29](#_Toc155547690)

[**Table S12.** Small for gestational age and microcephaly in 25,176 children of mothers with “**active**”****** epilepsy exposed and unexposed to antiseizure medication (ASM) in monotherapy during pregnancy 31](#_Toc155547691)

[**Table S13.** Complete case analyses of small for gestational age and microcephaly in 3,499,545 children exposed and unexposed to antiseizure medication (ASM) in monotherapy during pregnancy 32](#_Toc155547692)

[**Table S14.** Small for gestational age and microcephaly in 4,494,918 children exposed and unexposed to antiseizure medication (ASM) in monotherapy during pregnancy after restricting the exposure period to the period from LMP** to birth. 32](#_Toc155547693)

[**Table S15.** Head circumference and microcephaly in 4,299,375 children exposed and unexposed to antiseizure medication (ASM) after excluding 195,543 children with congenital malformations. 33](#_Toc155547694)

[**Table S16.** Small for gestational age and microcephaly for children from the overall population and in children of mothers with epilepsy exposed and unexposed to carbamazepine and lamotrigine in monotherapy during pregnancy. 34](#_Toc155547695)

[**Table S17.** Small for gestational age and microcephaly in 3,041,503 children with information of maternal body mass index (BMI) exposed and unexposed to antiseizure medication (ASM). 36](#_Toc155547696)

[**References** 38](#_Toc155547697)

# **List of investigators**

**Prenatal exposure to antiseizure medications and fetal growth: a population-based cohort study from the Nordic countries**

Jakob Christensen MD, PhD^1,2^; Helga Zoega, PhD^3,4^; Maarit K. Leinonen, MD, PhD^5^; Nils Erik Gilhus, MD, PhD^6,7^; Mika Gissler, PhD^5,8^; Jannicke Igland, PhD^9,10^; Yuelian Sun, PhD^1,11,12^; Torbjörn Tomson, MD, PhD^13^; Silje Alvestad, MD, PhD^6,14^; Marte-Helene Bjørk, MD, PhD^6,7^, and Julie Werenberg Dreier, PhD^6,11,15^

**Affiliations:**

1. Department of Neurology, Aarhus University Hospital, Aarhus, Denmark
2. Department of Clinical Medicine, Aarhus University, Aarhus, Denmark
3. Centre of Public Health Sciences, Faculty of Medicine, University of Iceland, Reykjavik, Iceland
4. School of Population Health, Faculty of Medicine and Health, UNSW Sydney, Sydney, Australia
5. Department of Knowledge Brokers, Finnish Institute for Health and Welfare, Helsinki, Finland
6. Department of Clinical Medicine, University of Bergen, Bergen, Norway
7. Department of Neurology, Haukeland University Hospital, Bergen, Norway
8. Region Stockholm, Academic Primary Health Care Centre, Stockholm, Sweden; Karolinska Institute, Department of Molecular Medicine and Surgery, Stockholm, Sweden
9. Department of Global Public Health and Primary Care, University of Bergen, Bergen, Norway
10. Department of Health and Caring Sciences, Western Norway University of Applied Sciences, Bergen, Norway
11. Centre for Integrated Register-based Research, CIRRAU, Aarhus University, Aarhus, Denmark
12. Department of Clinical Epidemiology, Aarhus University Hospital, Aarhus, Denmark
13. Department of Clinical Neuroscience, Karolinska Institutet, Stockholm, Sweden
14. National Center for Epilepsy, Oslo University Hospital, Oslo, Norway
15. National Centre for Register-Based Research, School of Business and Social Sciences, Aarhus University, Denmark

**ORCID numbers**

J Christensen: 0000-0002-9385-6435

H Zoega: 0000-0003-0761-9028

M K Leinonen: 0000-0002-7631-4749

J Cohen: 0000-0002-7300-0488

NE Gilhus: 0000-0001-6522-5102

M Gissler: 0000-0001-8254-7525

J Igland: 0000-0002-2289-0978

Y Sun: 0000-0002-6905-0503

T Tomson: 0000-0003-0554-5352

S Alvestad: 0000-0003-4655-8109

MH Bjørk: 0000-0002-5745-1094

J W Dreier: 0000-0002-9339-4170

# **E-methods**

Missing values for maternal education, maternal smoking in pregnancy, and head circumference were imputed using multiple imputation using chained equations (MICE)^1^ with ordinal logistic regression as imputation model for education, binary logistic regression for smoking, and linear regression for head circumference with 20 imputed datasets. All covariates used in the main analyses (country of birth, year of birth, sex of child, maternal age, parity, cohabitation, pre-pregnancy hospital admittances, maternal psychiatric disorders and use of psychotropic drugs in pregnancy, and maternal epilepsy) were included as imputation variables. In addition, we included gestational age at birth (in days), birth weight (in grams), a binary variable for low birth weight, a binary variable for small for gestational age (SGA), and a binary variable any antiseizure medications (ASM) exposure in pregnancy. We used the same 20 imputed datasets for all exposures and outcomes and obtained regression coefficients using Rubin’s rule.^2^

# **Figure S1.** Proportion and number of users of anti-seizure medication in pregnancy in Denmark, Finland, Norway, and Sweden by year of birth.

| **Table S1a** Data sources from five Nordic countries (Denmark, Finland, Iceland, Norway, and Sweden) | | |
| --- | --- | --- |
| **Register** | **Time period** ^a^ | **Description** |
| Medical birth registries (MBR) | Denmark 1997 | Information on births, term data, pregnancy length, maternal parity, smoking, maternal age, maternal comorbidity, and pregnancy- and birth complications. Reporting is compulsory for maternal- and neonatal wards. |
|  | Finland 1996 |  |
|  | Iceland 2004 |  |
|  | Norway 2005 |  |
|  | Sweden 2006 |  |
| Prescription drug registries (PDR) | Denmark 1995 | Data on all prescription fills from pharmacies including Anatomical therapeutical chemical (ATC) classification codes, drug strength, defined daily doses (DDD) in a package, package size and dispensing date. Indication for use (reimbursement codes) available in Norway, Denmark, and Finland. Over the counter medicines and medicines used during in-patient hospital stays are not included. In Finland, information is available for all prescriptions entitled to reimbursement. |
|  | Finland 1995 |  |
|  | Iceland 2003 |  |
|  | Norway 2004 |  |
|  | Sweden 2005 |  |
| National patient registries (NPR) | Denmark 1995 | Health administrative registries from private and public specialist care. Data on inpatient stays and outpatient care contacts included diagnoses coded according to International Statistical Classification of Diseases and Related Health Problems revision 10 (ICD-10).  In Finland, additional information concerning malformations in a separate malformation registry commencing in 1963. Since 2010 ambulatory care visits included. |
|  | Finland 1995 |  |
|  | Sweden 2005 |  |
|  | Norway 2008 |  |
|  | Iceland 2003 |  |
| National statistical institutes (NSI) | Cover all years included in the study | Socio-economic and demographic data on study participants in registers from Statistics Denmark, Statistics Norway, Statistics Finland, Statistics Sweden, and Statistics Iceland. Information on education, demographic data, emigration, and death. |
| a) The availability refers to the years of data available to the current study. | | |
| MBR: Medical birth register, NPR: National patient register, PDR: Prescribed drug register, NSI: National statistical institutes, ICD-10: International classification of diseases, revision 10, ATC: Anatomical therapeutical chemical classification, DDD: defined daily dose. | | |

| **Table S1b** Variables from five Nordic countries (Denmark, Finland, Iceland, Norway, and Sweden) | | | | | |
| --- | --- | --- | --- | --- | --- |
| **Cohort characteristic** | **Time Frame** | **Functional Form of Variable and Comments** |  |  | |
|  |  |  | **Register** | **ATC** | **ICD-10** |
| First day of last menstrual period (LMP) | n/a | Date of first day in last menstrual period: date of birth subtracted from gestational age at birth | MBR |  |  |
| Maternal epilepsy | Any time before child date of birth | Categorical: Y/N. At least one code of epilepsy from MBR (excl. DK) or NPR or code of ASM with epilepsy as indication or reason for reimbursement in PDR (available DK, FI, and NO) | NPR PDR MBR | N03A, N05BA09, S01EC01 | G40, G41 |
| Maternal active epilepsy | LMP-365 to birth | Categorical: Y/N. At least one code of epilepsy from MBR (excl. DK) or NPR or code of ASM with epilepsy as indication or reason for reimbursement in PDR (available DK, FI, and NO) | NPR PDR MBR | N03A, N05BA09, S01EC01 | G40, G41 |
| Any ASM in pregnancy | LMP-30 to birth | Categorical: Y/N  Any monotherapy or polytherapy | PDR | N03A, N05BA09, S01EC01 |  |
| ASM monotherapy in pregnancy | LMP-30 to birth | Categorical: Y/N  ≥1 prescriptions for a specific ASM and no prescriptions for any other ASM (18 distinct monotherapies) | PDR | N03A, N05BA09, S01EC01 |  |
| ASM mono- or polytherapy in pregnancy | LMP-30 to birth | Categorical: Y/N  ≥1 prescriptions for a specific ASM (27 distinct mono- or polytherapies) | PDR | N03A, N05BA09, S01EC01 |  |
| Country | At birth | Categorical: DK, FI, IS, NO, SE | MBR |  |  |
| Calendar year of birth | Year of birth | Categorical: 1996-1999, 2000-2004, 2005-2009, 2010-2014, 2015-2017 | MBR |  |  |
| Child sex | n/a | Categorical: Female or male | MBR |  |  |
| Maternal age | At birth | Categorical: <20, 20-24, 25-29, 30-34, 35-39, ≥40. | MBR |  |  |
| Maternal parity | n/a | Categorical: 0, 1, or ≥2  Number of previous deliveries: | MBR |  |  |
| Pre-pregnancy admittances | LMP-365 to LMP | Categorical: 0, 1, or ≥2  Number of pre-pregnancy hospital admittances. | NPR |  |  |
| Maternal Education | Year of birth | Ordinal (Compulsory, Secondary/Pre- university, Bachelor, Master/PhD) According to International classification for education (ISCED) | NSI |  |  |
| Smoking | At start of pregnancy | Categorical: Y/N.  Iceland: not available | MBR |  |  |
| Marital status | Year of birth | Categorical: Married/cohabitant or not | MBR |  |  |
| Maternal psychiatric disorder | Any time before child date of birth | Categorical: Y/N. | NPR |  | F00-F99 |
| Use of psychotropic drugs in pregnancy | LMP-30 to birth | Categorical: Y/N  Use of antidepressants, anxiolytics or antipsychotics | PDR | N06A, N05B (excl. N05BA09), N05A |  |
| **Birth outcome variables** | | | | | |
| Birth weight | At birth | Continuous, in gram (g) | MBR |  |  |
| Low birth weight | At birth | Categorical: Y/N  Birth weight <2,500 g | MBR |  |  |
| Small for gestational age | At birth | z-score for birth weight ≤10th percentile for gestational week, sex, and country. z-scores are calculated as z = (x-μ)/σ, where x is the raw score, μ is the population mean, and σ is the population standard deviation | MBR |  |  |
| Small for gestational age (alternative) | At birth | z-score for birth weight ≤3rd percentile for gestational week, sex, and country. z-scores are calculated as z = (x-μ)/σ, where x is the raw score, μ is the population mean, and σ is the population standard deviation | MBR |  |  |
| Head circumference | At birth | Continuous, in centimeters (cm) | MBR |  |  |
| Microcephaly | At birth | z-score for head circumference ≤3rd percentile for gestational week, sex, and country. z-scores are calculated as z = (x-μ)/σ, where x is the raw score, μ is the population mean, and σ is the population standard deviation | MBR |  |  |
|  | | | | | |
| **Outcome variables used to identify children excluded from the dataset (chromosomal abnormality) and children excluded in sensitivity analyses (major congenital malformations).** | | | | | |
| Chromosomal abnormality | Any time | Categorical: Y/N. Any of the codes listed | NPR MBR |  | Q90-Q99 |
| Major congenital malformations | Birth+365 days | Categorical: Y/N.  At least one code of major congenital  anomalies from NPR (DK, IS, NO, SE), MBR (DK, IS, NO, SE), CoDR (DK, NO), or MR (FI). * In accordance with Cohen, J.M., et al. (2023), Comparative Safety of Antiseizure Medication Monotherapy for Major Malformations. Ann Neurol, 93: 551-562^3^ | NPR MBR  MR  CoDR |  | Q00-Q89* |
|  |  |  |  |  |  |
| LMP: first day of the last menstrual period, MBR: Medical birth register, NPR: National patient register, PDR: Prescribed drug register, NSI: National statistical institutes, MR: Malformation Register, CoDR: Cause of Death Register, ICD-10: International classification of diseases, revision 10. ATC: anatomical therapeutic chemical classification system. FI: Finland, DK: Denmark, IS: Iceland, NO: Norway, SE: Sweden. | | | | | |

| **Table S1c:** Ethical and/or data protection authorities that have approved the project in all countries | | | | |
| --- | --- | --- | --- | --- |
| **Country** | **Ethical approval** | **Data protection approvals** | **Data transfer agreement*** | **Data protection agreement** |
| **Denmark** | Not required for register-based research | Data protection agency |  | x |
| **Finland** | Not required for register-based research | Local data protection officer | x | x |
| **Iceland** | x | National Bioethics Committee, National Data Protection Authority | x | x |
| **Norway** | x | Data protection impact assessment  Norwegian Data Protection Authority | x | x |
| **Sweden** | x | Local data protection officer | x | x |
| *The data were analyzed in Denmark; thus, a data transfer agreement was not relevant. | | | | |

| **Table S2.** Unadjusted and adjusted estimates of birth weight, low birth weight (<2,500 grams), and small for gestational age in 4,494,918 children exposed and unexposed to antiseizure medication (ASM) in monotherapy during pregnancy | | | | | | | | | |
| --- | --- | --- | --- | --- | --- | --- | --- | --- | --- |
|  | *Exposed* | **Birth weight (gram)** | |  | **Low birth weight** | |  | **Small for gestational age** | |
| Exposure groups | *n* | Unadjusted difference  (95% CI) | Adjusted difference  (95% CI) |  | Unadjusted OR (95% CI) | Adjusted OR (95% CI) |  | Unadjusted OR  (95% CI) | Adjusted OR  (95% CI) |
| No ASM | 4,467,848 | 0·00 (ref) | 0·00 (ref) |  | 1·00 (ref) | 1·00 (ref) |  | 1·00 (ref) | 1·00 (ref) |
| Any ASM | 27,070 | -95 (-102 to -89) | -26 (-33 to -18) |  | 1·72 (1·63-1·81) | 1·14 (1·07-1·22) |  | 1·33 (1·28-1·38) | 1·13 (1·08-1·18) |
| **Monotherapies** |  |  |  |  |  |  |  |  |  |
| Lamotrigine | 8,756 | -54 (-65 to -42) | 26 (14 to 38) |  | 1·45 (1·31-1·60) | 0·94 (0·84-1·05) |  | 1·06 (0·99-1·13) | 0·91 (0·85-0·99) |
| Carbamazepine | 3,424 | -86 (-104 to -67) | -58 (-77 to -38) |  | 1·88 (1·63-2·17) | 1·46 (1·25-1·71) |  | 1·29 (1·16-1·42) | 1·25 (1·12-1·40) |
| Valproate | 2,669 | -37 (-58 to -16) | 30 (9 to 51) |  | 1·44 (1·20-1·73) | 1·00 (0·83-1·21) |  | 1·25 (1·11-1·40) | 1·07 (0·95-1·21) |
| Pregabalin | 2,214 | -159 (-182 to -136) | -58 (-81 to -35) |  | 2·00 (1·68-2·38) | 1·23 (1·03-1·47) |  | 1·51 (1·34-1·70) | 1·16 (1·02-1·31) |
| Oxcarbazepine | 1,591 | -88 (-115 to -61) | -39 (-67 to -12) |  | 1·60 (1·27-2·00) | 1·23 (0·97-1·55) |  | 1·61 (1·40-1·85) | 1·48 (1·28-1·71) |
| Clonazepam | 1,358 | -179 (-208 to -149) | -63 (-93 to -34) |  | 2·07 (1·66-2·57) | 1·05 (0·84-1·33) |  | 1·75 (1·52-2·02) | 1·27 (1·10-1·48) |
| Gabapentin | 1,336 | -144 (-174 to -114) | -88 (-117 to -59) |  | 1·85 (1·46-2·33) | 1·25 (0·98-1·59) |  | 1·30 (1·10-1·52) | 1·13 (0·96-1·34) |
| Levetiracetam | 1,077 | -85 (-118 to -52) | -29 (-62 to 4) |  | 1·49 (1·12-1·98) | 1·12 (0·84-1·51) |  | 1·18 (0·98-1·43) | 1·08 (0·89-1·32) |
| Topiramate | 638 | -114 (-157 to -72) | -66 (-108 to -24) |  | 2·05 (1·49-2·82) | 1·51 (1·09-2·10) |  | 1·60 (1·28-1·98) | 1·48 (1·18-1·85) |
| Phenobarbital | 183 | -193 (-273 to -113) | -144 (-223 to -65) |  | 2·54 (1·47-4·37) | 1·52 (0·86-2·70) |  | 1·70 (1·14-2·52) | 1·46 (0·97-2·20) |
| Acetazolamide | 127 | -109 (-205 to -13) | -43 (-137 to 50) |  | 0·74 (0·24-2·33) | NA |  | 1·21 (0·70-2·07) | 1·00 (0·58-1·73) |
| Phenytoin | 81 | 62 (-58 to 182) | 76 (-44 to 195) |  | 2·45 (1·07-5·62) | 1·94 (0·83-4·51) |  | 0·85 (0·39-1·85) | 0·79 (0·36-1·73) |
| Clobazam | 44 | -157 (-320 to 7) | -126 (-285 to 33) |  | 4·83 (2·04-11·43) | 3·65 (1·52-8·76) |  | 1·16 (0·46-2·93) | 1·14 (0·44-2·92) |
| Primidone | 34 | -141 (-327 to 45) | -120 (-301 to 61) |  | NA | NA |  | 1·93 (0·80-4·66) | 1·84 (0·74-4·55) |
| Zonisamide | 19 | -185 (-434 to 63) | -90 (-332 to 152) |  | NA | NA |  | NA | NA |
| Vigabatrin | 17 | -18 (-281 to 245) | 46 (-211 to 302) |  | NA | NA |  | NA | NA |
| Ethosuximide | 11 | -251 (-577 to 76) | -170 (-488 to 148) |  | NA | NA |  | NA | NA |
| Lacosamide | 9 | 31 (-330 to 393) | 134 (-239 to 506) |  | NA | NA |  | NA | NA |
| NA = Not analyzed due to low numbers.  ND = not analyzed | | | | | | | | | |
| Adjustment: Country of birth, year of birth, sex of child, maternal age, parity, cohabitation, pre-pregnancy hospital admittances, maternal education, smoking in pregnancy, maternal psychiatric disorders, maternal epilepsy, and use of psychotropic drugs in pregnancy. Due to the size of the dataset, we did not account for siblings born to the same mother. Using robust standard errors to account for siblings only had minor impact on the confidence intervals. | | | | | | | | | |

| **Table S3.** Small for gestational age (SGA) by 3rd percentile in 4,494,918 children from the overall population and in the subset of 38,714 children of mothers with epilepsy prenatally exposed and unexposed to antiseizure medication (ASM) in monotherapy during pregnancy | | | | | | | | | |
| --- | --- | --- | --- | --- | --- | --- | --- | --- | --- |
|  | **All Children** | | | |  | **Children of mothers with epilepsy** | | | |
|  | Exposed |  | **Small for gestational age**  **(≤ 3rd percentile)** | |  | Exposed |  | **Small for gestational age**  **(≤ 3rd percentile)** | |
| Exposure groups | n |  | N (%) | Adjusted OR  (95% CI) |  | *n* |  | N (%) | Adjusted OR  (95% CI) |
| No ASM | 4,467,848 |  | 133,732 (3·0) | 1·00 (ref) |  | 22,227 |  | 727 (3·3) | 1·00 (ref)· |
| Any ASM | 27,070 |  | 1,140 (4·2) | 1·14 (1·06-1·23) |  | 16,487 |  | 697 (4·2) | 1·19 (1·06-1·34) |
|  |  |  |  |  |  |  |  |  |  |
| **Monotherapies** |  |  |  |  |  |  |  |  |  |
| Lamotrigine | 8,756 |  | 274 (3·1) | 0·88 (0·77-1·00) |  | 5,299 |  | 169 (3·2) | 0·95 (0·80-1·13) |
| Carbamazepine | 3,424 |  | 139 (4·1) | 1·29 (1·07-1·55) |  | 2,669 |  | 111 (4·2) | 1·34 (1·06-1·70) |
| Valproate | 2,669 |  | 102 (3·8) | 1·02 (0·83-1·26) |  | 1,953 |  | 76 (3·9) | 1·13 (0·86-1·50) |
| Pregabalin | 2,214 |  | 114 (5·1) | 1·26 (1·03-1·52) |  | 120 |  | 6 (5·0) | 1·14 (0·49-2·65) |
| Oxcarbazepine | 1,591 |  | 91 (5·7) | 1·77 (1·41-2·21) |  | 1,462 |  | 85 (5·8) | 1·73 (1·30-2·31) |
| Clonazepam | 1,358 |  | 85 (6·3) | 1·36 (1·08-1·71) |  | 339 |  | 19 (5·6) | 1·29 (0·79-2·10) |
| Gabapentin | 1,336 |  | 47 (3·5) | 0·97 (0·72-1·31) |  | 138 |  | 5 (3·6) | 0·91 (0·37-2·26) |
| Levetiracetam | 1,077 |  | 42 (3·9) | 1·24 (0·90-1·72) |  | 1,063 |  | 41 (3·9) | 1·26 (0·90-1·76) |
| Topiramate | 638 |  | 32 (5·0) | 1·57 (1·10-2·26) |  | 290 |  | 14 (4·8) | 1·47 (0·85-2·55) |
| Phenobarbital | 183 |  | 13 (7·1) | 1·96 (1·11-3·49) |  | 47 |  | <5 | NA |
| Acetazolamide | 127 |  | <5 | NA |  | 9 |  | <5 | NA |
| Phenytoin | 81 |  | 7^a^ (-) | 1·89 (0·76-4·75) |  | 64 |  | 7^a^ (-) | 2·10 (0·81-5·44) |
| Clobazam | 44 |  | <5 | NA |  | 27 |  | <5 | NA |
| Primidone | 34 |  | <5 | NA |  | 27 |  | <5 | NA |
| Zonisamide | 19 |  | <5 | NA |  | 19 |  | <5 | NA |
| Vigabatrin | 17 |  | <5 | NA |  | 9 |  | <5 | NA |
| Ethosuximide | 11 |  | <5 | NA |  | 11 |  | <5 | NA |
| Lacosamide | 9 |  | <5 | NA |  | 9 |  | 727 (3·3) | NA |
| NA = Not analyzed due to low numbers.  ^a^ Number is copied from Table 2a/2b for data privacy reasons, as differences are <5. However, analyses are based on actual numbers.  Adjustment: Basic adjustment and maternal age, parity, cohabitation, pre-pregnancy hospital admittances, maternal education, smoking in pregnancy, maternal psychiatric disorders, maternal epilepsy, and use of psychotropic drugs in pregnancy. | | | | | | | | | |

| **Table S4.** Unadjusted and adjusted estimates of head circumference and microcephaly in 4,494,918 children exposed and unexposed to antiseizure medication (ASM) in monotherapy during pregnancy | | | | | | |
| --- | --- | --- | --- | --- | --- | --- |
|  | *Exposed* | **Head circumference (cm)** | |  | **Microcephaly** | |
| Exposure groups | *n* | Unadjusted difference  (95% CI) | Adjusted  difference  (95% CI) |  | Unadjusted  OR  (95% CI) | Adjusted  OR  (95% CI) |
| No ASM | 4,467,848 | 0·00 (ref) | 0·00 (ref) |  | 1·00 (ref) | 1·00 (ref) |
| Any ASM | 27,070 | -0·23 (-0·25 to -0·21) | -0·06 (-0·08 to -0·04) |  | 1·23 (1·15-1 ·32) | 1·09 (1·01-1·18) |
|  |  |  |  |  |  |  |
| **Monotherapies** |  |  |  |  |  |  |
| Lamotrigine | 8,756 | -0·14 (-0·18 to -0·11) | 0·06 (0·03 to 0·10) |  | 1·05 (0·93-1·18) | 0·94 (0·83-1·07) |
| Carbamazepine | 3,424 | -0·29 (-0·35 to -0·22) | -0·27 (-0·33 to -0·20) |  | 1·32 (1·09-1·61) | 1·43 (1·17-1·75) |
| Valproate | 2,669 | -0·11 (-0·18 to -0·04) | 0·03 (-0·04 to 0·09) |  | 1·22 (0·97-1·52) | 1·08 (0·85-1·37) |
| Pregabalin | 2,214 | -0·39 (-0·46 to -0·32) | -0·09 (-0·16 to -0·02) |  | 1·26 (1·01-1·58) | 1·01 (0·80-1·27) |
| Oxcarbazepine | 1,591 | -0·11 (-0·20 to -0·02) | -0·03 (-0·12 to 0·06) |  | 1·08 (0·80-1·46) | 1·02 (0·75-1·39) |
| Clonazepam | 1,358 | -0·42 (-0·51 to -0·32) | -0·15 (-0·25 to -0·06) |  | 1·52 (1·16-1·99) | 1·15 (0·88-1·51) |
| Gabapentin | 1,336 | -0·23 (-0·32 to -0·14) | -0·10 (-0·19 to -0·01) |  | 0·80 (0·56-1·14) | 0·67 (0·46-0·97) |
| Levetiracetam | 1,077 | -0·12 (-0·22 to -0·02) | 0·02 (-0·08 to 0·12) |  | 0·98 (0·68-1·40) | 0·89 (0·61-1·30) |
| Topiramate | 638 | -0·19 (-0·33 to -0·06) | -0·05 (-0·18 to 0·08) |  | 1·46 (0·99-2·14) | 1·35 (0·92-1·99) |
| Phenobarbital | 183 | -0·49 (-0·75 to -0·24) | -0·45 (-0·69 to -0·20) |  | 2·16 (1·17-3·96) | 1·82 (0·96-3·47) |
| Acetazolamide | 127 | 0·01 (-0·29 to 0·31) | 0·15 (-0·14 to 0·44) |  | NA | NA |
| Phenytoin | 81 | 0·21 (-0·20 to 0·62) | 0·12 (-0·29 to 0·54) |  | NA | NA |
| Clobazam | 44 | -0·20 (-0·71 to 0·32) | -0·19 (-0·69 to 0·31) |  | NA | NA |
| Primidone | 34 | -0·41 (-0·99 to 0·17) | -0·45 (-1·02 to 0·11) |  | NA | NA |
| Zonisamide | 19 | -0·83 (-1·59 to -0·06) | -0·61 (-1·35 to 0·13) |  | NA | NA |
| Vigabatrin | 17 | 0·74 (-0·08 to 1·56) | 0·82 (0·03 to 1·62) |  | NA | NA |
| Ethosuximide | 11 | -0·65 (-1·65 to 0·36) | -0·38 (-1·36 to 0·59) |  | NA | NA |
| Lacosamide | 9 | 0·43 (-0·68 to 1·55) | 0·79 (-0·35 to 1·93) |  | NA | NA |
| NA = Not analyzed due to low numbers. | | | | | | |
| Adjustment: Country of birth, year of birth, sex of child, maternal age, parity, cohabitation, pre-pregnancy hospital admittances, maternal education, smoking in pregnancy, maternal psychiatric disorders, maternal epilepsy, and use of psychotropic drugs in pregnancy. Due to the size of the dataset, we did not account for siblings born to the same mother. Using robust standard errors to account for siblings only had minor impact on the confidence intervals. | | | | | | |

| **Table S5.** Small for gestational age (SGA) and microcephaly in 4,494,918 children exposed and unexposed to high- and low-dose antiseizure medication (ASM) in monotherapy during pregnancy | | | | | | | |
| --- | --- | --- | --- | --- | --- | --- | --- |
|  | *Exposed* |  | **Small for gestational age** | |  | **Microcephaly** | |
|  | *n* |  | *N (%)* | Adjusted OR |  | *N* (%)* | Adjusted OR |
| Reference | 4,467,850 |  | 446,270 (10·0) | 1·00 (ref) |  | 134,023 (3·0) | 1·00 (ref) |
| **Monotherapies** |  |  |  |  |  |  |  |
| Lamotrigine |  |  |  |  |  |  |  |
| Low dose <150 mg | 3,760 |  | 430 (11·3) | 0·93 (0·83-1·03) |  | 138 (3·7) | 1·02 (0·86-1·23) |
| High dose ≥150 mg | 5,000 |  | 490 (9·9) | 0·90 (0·82-1·00) |  | 136 (2·7) | 0·86 (0·72-1·03) |
|  |  |  |  |  |  |  |  |
| Carbamazepine |  |  |  |  |  |  |  |
| Low dose <500 mg | 1,300 |  | 160 (12·4) | 1·16 (0·98-1·38) |  | 46 (3·6) | 1·19 (0·85-1·67) |
| High dose ≥500 mg | 2,130 |  | 270 (12·6) | 1·32 (1·15-1·51) |  | 89 (4·2) | 1·60 (1·25-2·06) |
|  |  |  |  |  |  |  |  |
| Valproate |  |  |  |  |  |  |  |
| Low dose < 750 mg | 1,370 |  | 160 (11·8) | 0·98 (0·83-1·16) |  | 46 (3·4) | 0·93 (0·67-1·29) |
| High dose ≥750 mg | 1,300 |  | 160 (12·6) | 1·18 (1·00-1·40) |  | 51 (3·9) | 1·26 (0·92-1·74) |
|  |  |  |  |  |  |  |  |
| Pregabalin |  |  |  |  |  |  |  |
| Low dose <150 mg | 1,550 |  | 220 (14·1) | 1·18 (1·01-1·36) |  | 60 (3·9) | 1·05 (0·80-1·38) |
| High dose ≥150 mg | 670 |  | 100 (15·0) | 1·12 (0·90-1-39) |  | 24 (3·6) | 0·91 (0·59-1·41) |
|  |  |  |  |  |  |  |  |
| Oxcarbazepine |  |  |  |  |  |  |  |
| Low dose <500 mg | 290 |  | 30 (9·7) | 0·85 (0·57-1·26) |  | <5 | NA |
| High dose ≥500 mg | 1,300 |  | 210 (16·4) | 1·64 (1·41-1·92) |  | 50** (3·7) | 1·19 (0·87-1·63) |
|  |  |  |  |  |  |  |  |
| Clonazepam |  |  |  |  |  |  |  |
| Low dose <4 mg | 1,200 |  | 170 (14·4) | 1·12 (0·95-1·32) |  | 49 (4·1) | 1·07 (0·79-1·45) |
| High dose ≥4 mg | 150 |  | 50 (31·2) | 2·66 (1·86-3·81) |  | 12 (7·8) | 1·72 (0·88-3·35) |
|  |  |  |  |  |  |  |  |
| Gabapentin |  |  |  |  |  |  |  |
| Low dose <900 mg | 1,110 |  | 130 (12·0) | 1·10 (0·91-1·32) |  | 23 (2·1) | 0·57 (0·37-0·89) |
| High dose ≥900 mg | 220 |  | 40 (15·6) | 1·29 (0·89-1·87) |  | 10 (4·5) | 1·11 (0·55-2·25) |
|  |  |  |  |  |  |  |  |
| Levetiracetam |  |  |  |  |  |  |  |
| Low dose <750 mg | 240 |  | 40 (15·3) | 1·51 (1·04-2·17) |  | 9 (3·8) | 1·20 (0·59-2·45) |
| High dose ≥750 mg | 840 |  | 90 (10·6) | 0·97 (0·77-1·22) |  | 23 (2·7) | 0·81 (0·52-1·26) |
|  |  |  |  |  |  |  |  |
| Topiramate |  |  |  |  |  |  |  |
| Low dose <150 mg | 520 |  | 70 (13·4) | 1·30 (1·00-1·68) |  | 20 (3·9) | 1·22 (0·78-1·91) |
| High dose ≥150 mg | 120 |  | 30 (22·3) | 2·34 (1·50-3·65) |  | 7 (5·8) | 1·95 (0·90-4·22) |
| NA = Not analyzed due to low numbers. | | | | | | | |
| Adjustment: Country of birth, year of birth, sex of child, maternal age, parity, cohabitation, pre-pregnancy hospital admittances, maternal education, smoking in pregnancy, maternal psychiatric disorders, maternal epilepsy, and use of psychotropic drugs in pregnancy. | | | | | | | |
| *Rounded average of imputed Ns. | | | | | | | |
| **Rounded to nearest 10. | | | | | | | |

# **Tables S6 and S7** Risk of restricted intrauterine growth in 4,456,204 children of women without epilepsy prenatally exposed and unexposed to ASMs.

In children of women without epilepsy, prenatal exposure to pregabalin (aOR: 1·17 (95% CI: 1·03-1·32)) and clonazepam (aOR: 1·22 (95% CI: 1·02-1·45)) was associated with small for gestational age **(Table S6**), but no prenatal exposure to ASMs was associated with risk of microcephaly **(Table S7)**.

| **Table S6.** Birth weight, low birth weight (<2,500 g), and small for gestational age (SGA) in 4,456,204 children of **mothers without epilepsy** exposed and unexposed to antiseizure medication (ASM) in monotherapy during pregnancy | | | | | | | | | | | | | | | | | | |
| --- | --- | --- | --- | --- | --- | --- | --- | --- | --- | --- | --- | --- | --- | --- | --- | --- | --- | --- |
|  | | *Exposed* |  | **Birth weight (gram)** | | | | |  | **Low birth weight (<2,500 g)** | | | | |  | **Small for gestational age** | | |
| Exposure groups | | *n* |  | *Mean (s.d.)* | | Adjusted mean difference  (95% CI) | | |  | *N (%)* | | Adjusted OR  (95% CI) | | |  | *N (%)* | | Adjusted OR  (95% CI) |
| No ASM | | 4,445,621 |  | 3,536 (553) | | 0·00 (ref) | | |  | 140,436 (3·2) | | 1·00 (ref) | | |  | 443,851 (10·0) | | 1·00 (ref) |
| Any ASM | | 10,583 |  | 3,416 (590) | | -32 (-43 to -22) | | |  | 581 (5·5) | | 1·12 (1·02-1·22) | | |  | 1,361 (12·9) | | 1·06 (1·00-1·13) |
|  | |  |  |  | |  | | |  |  | |  | | |  |  | |  |
| **Monotherapies** | |  |  |  | |  | | |  |  | |  | | |  |  | |  |
| Lamotrigine | | 3,457 |  | 3,457 (591) | | 23 (5 to 41) | | |  | 165 (4·8) | | 0·93 (0·79-1·09) | | |  | 374 (10·8) | | 0·88 (0·79-0·99) |
| Carbamazepine | | 755 |  | 3,454 (616) | | -50 (-89 to -11) | | |  | 45 (6·0) | | 1·53 (1·12-2·10) | | |  | 87 (11·5) | | 1·01 (0·80-1·28) |
| Valproate | | 716 |  | 3,432 (584) | | -7 (-48 to 33) | | |  | 28 (3·9) | | 0·81 (0·55-1·19) | | |  | 96 (13·4) | | 1·07 (0·86-1·34) |
| Pregabalin | | 2,094 |  | 3,380 (583) | | -59 (-82 to -35) | | |  | 128 (6·1) | | 1·25 (1·04-1·51) | | |  | 303 (14·5) | | 1·17 (1·03-1·32) |
| Oxcarbazepine | | 129 |  | 3,408 (532) | | -86 (-179 to 8) | | |  | 8 (6·2) | | 1·56 (0·76-3·23) | | |  | 19 (14·7) | | 1·31 (0·80-2·15) |
| Clonazepam | | 1,019 |  | 3,367 (567) | | -51 (-85 to -18) | | |  | 59 (5·8) | | 0·96 (0·73-1·28) | | |  | 164 (16·1) | | 1·22 (1·02-1·45) |
| Gabapentin | | 1,198 |  | 3,393 (597) | | -92 (-123 to -61) | | |  | 68 (5·7) | | 1·28 (1·00-1·66) | | |  | 153 (12·8) | | 1·16 (0·97-1·38) |
| Levetiracetam | | 14 |  | 2,980 (740) | | -587 (-869 to -306) | | |  | <5 | | NA | | |  | <5 | | NA |
| Topiramate | | 348 |  | 3,465 (592) | | -33 (-90 to 24) | | |  | 19 (5·5) | | 1·39 (0·87-2·21) | | |  | 43 (12·4) | | 1·19 (0·86-1·64) |
| Phenobarbital | | 136 |  | 3,338 (616) | | -143 (-235 to -52) | | |  | 13 (9·6) | | 1·90 (1·03-3·49) | | |  | 22 (16·2) | | 1·42 (0·89-2·26) |
| Acetazolamide | | 118 |  | 3,439 (503) | | -39 (-137 to 58) | | |  | <5 | | NA | | |  | 13 (11·0) | | 0·95 (0·53-1·69) |
| Phenytoin | | 17 |  | 3,814 (613) | | 169 (-95 to 433) | | |  | <5 | | NA | | |  | 0 | | NA |
| Clobazam | | 17 |  | 3,426 (820) | | -103 (-359 to 153) | | |  | <5 | | NA | | |  | <5 | | NA |
| Primidone | | 7 |  | 3,485 (542) | | -71 (-470 to 328) | | |  | <5 | | NA | | |  | 0 | | NA |
| Zonisamide | | 0 |  | NA | | NA | | |  | NA | | NA | | |  | NA | | NA |
| Vigabatrin | | 8 |  | 3,374 (516) | | -164 (-538 to 211) | | |  | <5 | | NA | | |  | <5 | | NA |
| Ethosuximide | | 0 |  | NA | | NA | | |  | NA | | NA | | |  | NA | | NA |
| Lacosamide | | 0 |  | NA | | NA | | |  | NA | | NA | | |  | NA | | NA |
| NA = Not analyzed due to low numbers. | | | | | | | | | | | | | | | | | | |
| Adjustment: Country of birth, year of birth, sex of child, maternal age, parity, cohabitation, pre-pregnancy hospital admittances, maternal education, smoking in pregnancy, maternal psychiatric disorders, maternal epilepsy, and use of psychotropic drugs in pregnancy. | | | | | | | | | | | | | | | | | | |
| **Table S7.** Head circumference and microcephaly in 4,456,204 children of **mothers without epilepsy** exposed and unexposed to antiseizure medication (ASM) in monotherapy during pregnancy | | | | | | | | | | | | | | | | | |  |
|  | | | | *Exposed* | |  | **Head circumference, cm** | | | | |  | **Microcephaly** | | | | |  |
| Exposure groups | | | | *n* | |  | *Mean (s.d.)* | | | Adjusted mean difference (cm) (95% CI) | |  | *N* (%)* | | | Adjusted OR  (95% CI) | |  |
| No ASM | | | | 4,445,621 | |  | 35·0 (1·8) | | | 0·00 (ref) | |  | 133,324 (3·0) | | | 1·00 (ref) | |  |
| Any ASM | | | | 10,583 | |  | 34·7 (1·8) | | | -0·05 (-0·09 to -0·02) | |  | 404 (3·8) | | | 1·04 (0·94-1·16) | |  |
|  | | | |  | |  |  | | |  | |  |  | | |  | |  |
| **Monotherapies** | | | |  | |  |  | | |  | |  |  | | |  | |  |
| Lamotrigine | | | | 3,457 | |  | 34·8 (1·9) | | | 0·05 (-0·01 to 0·10) | |  | 124 (3·6) | | | 1·00 (0·84-1·21) | |  |
| Carbamazepine | | | | 755 | |  | 34·8 (2·1) | | | -0·19 (-0·32 to -0·06) | |  | 30 (4·0) | | | 1·22 (0·80-1·86) | |  |
| Valproate | | | | 716 | |  | 34·8 (1·8) | | | -0·02 (-0·15 to 0·11) | |  | 30 (4·1) | | | 1·07 (0·69-1·66) | |  |
| Pregabalin | | | | 2,094 | |  | 34·6 (1·8) | | | -0·09 (-0·16 to -0·02) | |  | 79 (3·8) | | | 1·02 (0·80-1·28) | |  |
| Oxcarbazepine | | | | 129 | |  | 35·0 (1·9) | | | -0·00 (-0·32 to 0·32) | |  | <5 | | | NA | |  |
| Clonazepam | | | | 1,019 | |  | 34·6 (1·9) | | | -0·14 (-0·25 to -0·03) | |  | 52 (5·1) | | | 1·26 (0·94-1·69) | |  |
| Gabapentin | | | | 1,198 | |  | 34·8 (1·8) | | | -0·09 (-0·19 to 0·00) | |  | 30 (2·5) | | | 0·69 (0·47-1·02) | |  |
| Levetiracetam | | | | 14 | |  | 33·9 (2·8) | | | -1·20 (-2·08 to -0·32) | |  | <5 | | | NA | |  |
| Topiramate | | | | 348 | |  | 34·8 (1·7) | | | -0·00 (-0·18 to 0·17) | |  | 16 (4·6) | | | 1·42 (0·86-2·35) | |  |
| Phenobarbital | | | | 136 | |  | 34·6 (1·9) | | | -0·39 (-0·68 to -0·10) | |  | 8 (6·0) | | | 1·57 (0·72-3·40) | |  |
| Acetazolamide | | | | 118 | |  | 35·1 (1·5) | | | 0·17 (-0·13 to 0·47) | |  | 0 | | | NA | |  |
| Phenytoin | | | | 17 | |  | 35·7 (1·9) | | | 0·36 (-0·60 to 1·32) | |  | 0 | | | NA | |  |
| Clobazam | | | | 17 | |  | 35·0 (2·0) | | | -0·08 (-0·91 to 0·74) | |  | 0 | | | NA | |  |
| Primidone | | | | 7 | |  | 35·0 (1·7) | | | -0·15 (-1·37 to 1·07) | |  | 0 | | | NA | |  |
| Zonisamide | | | | 0 | |  | NA | | | NA | |  | NA | | | NA | |  |
| Vigabatrin | | | | 8 | |  | 35·0 (2·1) | | | 0·42 (-0·77 to 1·60) | |  | <5 | | | NA | |  |
| Ethosuximide | | | | 0 | |  | NA | | | NA | |  | NA | | | NA | |  |
| Lacosamide | | | | 0 | |  | NA | | | NA | |  | NA | | | NA | |  |
| NA = Not analyzed due to low numbers. | | | | | | | | | | | | | | | | | |  |
| Adjustment: Country of birth, year of birth, sex of child, maternal age, parity, cohabitation, pre-pregnancy hospital admittances, maternal education, smoking in pregnancy, maternal psychiatric disorders, maternal epilepsy, and use of psychotropic drugs in pregnancy. | | | | | | | | | | | | | | | | | |  |
| *Rounded average of 20 imputed Ns. | | | | | | | | | | | | | | | | | |  |

# **Tables S8-S11** Risk of restricted intrauterine growth in children after prenatal exposure to ASMs in mono- and polytherapy combined.

In analyses including children exposed to ASMs in mono- and polytherapy (i.e., not restricting to monotherapy), small for gestational age was associated with exposure to carbamazepine (aOR: 1·35 (95% CI: 1·22-1·49)), valproate (aOR: 1·12 (95% CI: 1·01-1·25)), pregabalin (aOR: 1·17 (95% CI: 1·05-1·32)), oxcarbazepine (aOR: 1·80 (95% CI: 1·60-2·03)), clonazepam (aOR: 1·30 (95% CI: 1·15-1·47)), gabapentin (aOR: 1·19 (95% CI: 1·02-1·38)), levetiracetam 1·30 (95% CI: 1·14-1·49)), topiramate (aOR: 1·70 (95% CI: 1·44-2·01)), and clobazam (aOR: 1·45 (95% CI: 1·09-1·94) **(Table S8)**.

Similar associations were observed when we restricted analyses to children of women with epilepsy, albeit no associations were identified for valproate, gabapentin, and pregabalin **(Table S9).** Additional analyses of other drugs used in mono- and polytherapy are shown in **Tables S8-S9**).

In analyses of microcephaly, prenatal exposure to ASMs in mono- and polytherapy identified an association between microcephaly and prenatal exposure to carbamazepine (aOR: 1·50 (95% CI: 1·25-1·79)), valproate (aOR: 1·22 (95% CI: 1·00-1·48), levetiracetam (aOR: 1·28 (95% CI: 1·01-1·62), cloabazam (aOR: 1·82 (95% CI: 1·16-2·88), primidone (aOR: 3·17 (95% CI: 1·24-8·07) and zonisamide (aOR: 3·66 (95% CI: 1·98-6·76) **(Table S10)**. Similar associations between prenatal exposure to ASMs in mono- and polytherapy and microcephaly were found for the same ASMs when restricting analyses to children of women with epilepsy, albeit no association was found for prenatal exposure to levetiracetam **(Table S11)**. Additional analyses of other drugs used in mono- and polytherapy are shown in **Tables S10-S11)**.

| **Table S8**. Birth weight, low birth weight (<2,500 g), and small for gestational age (SGA) in 4,494,918 children exposed and unexposed to antiseizure medication (ASM) in **mono- and polytherapy** during pregnancy | | | | | | | | | | |
| --- | --- | --- | --- | --- | --- | --- | --- | --- | --- | --- |
|  | *Exposed* |  | **Birth weight**  **(g)** | |  | **Low birth weight**  **(<2,500 g)** | |  | **Small for gestational age (SGA)** | |
| Exposure groups | *n* |  | *Mean (s.d.)* | Adjusted mean difference (95% CI) |  | *N (%)* | Adjusted OR  (95% CI) |  | *N (%)* | Adjusted OR  (95% CI) |
| No ASM | 4,467,848 |  | 3,536 (553) | 0·00 (ref) |  | 141,442 (3.2) | 1·00 (ref) |  | 446,267 (10.0) | 1·00 (ref) |
| Any ASM | 27,070 |  | 3,441 (600) | -26 (-33 to -18) |  | 1,440 (5.3) | 1·14 (1·07-1·22) |  | 3,479 (12.9) | 1·13 (1·08-1·18) |
|  |  |  |  |  |  |  |  |  |  |  |
| **Mono- and polytherapy** |  |  |  |  |  |  |  |  |  |  |
| Lamotrigine | 10,609 |  | 3,473 (592) | 17 (6 to 29) |  | 489 (4.6) | 0·95 (0·86-1·05) |  | 1,168 (11.0) | 0·96 (0·90-1·03) |
| Carbamazepine | 4,171 |  | 3,430 (630) | -72 (-90 to -55) |  | 257 (6.2) | 1·51 (1·31-1·74) |  | 565 (13.5) | 1·35 (1·22-1·49) |
| Valproate | 3,638 |  | 3,478 (619) | 13 (-6 to 32) |  | 188 (5.2) | 1·13 (0·96-1·32) |  | 465 (12.8) | 1·12 (1·01-1·25) |
| Pregabalin | 2,586 |  | 3,367 (589) | -60 (-81 to -38) |  | 165 (6.4) | 1·22 (1·03-1·44) |  | 379 (14.7) | 1·17 (1·05-1·32) |
| Oxcarbazepine | 2,145 |  | 3,396 (600) | -86 (-110 to -62) |  | 129 (6.0) | 1·43 (1·18-1·74) |  | 385 (17.9) | 1·80 (1·60-2·03) |
| Clonazepam | 2,039 |  | 3,348 (589) | -73 (-97 to -49) |  | 143 (7.0) | 1·20 (1·00-1·44) |  | 332 (16.3) | 1·30 (1·15-1·47) |
| Gabapentin | 1,556 |  | 3,383 (587) | -90 (-117 to -63) |  | 88 (6) | 1·20 (0·96-1·50) |  | 207 (13.3) | 1·19 (1·02-1·38) |
| Levetiracetam | 2,098 |  | 3,424 (574) | -53 (-77 to -29) |  | 99 (5) | 1·11 (0·89-1·37) |  | 287 (13.7) | 1·30 (1·14-1·49) |
| Topiramate | 1,045 |  | 3,386 (598) | -83 (-116 to -49) |  | 71 (7) | 1·49 (1·16-1·92) |  | 186 (17.8) | 1·70 (1·44-2·01) |
| Phenobarbital | 228 |  | 3,350 (590) | -123 (-193 to -52) |  | 14^a^ (-) | 1·39 (0·83-2·33) |  | 34 (15) | 1·31 (0·90-1·90) |
| Acetazolamide | 127^a^ |  | 3,419 (514) | -50 (-142 to 43) |  | <5 | NA |  | 15 (12) | 0·98 (0·57-1·69) |
| Phenytoin | 152 |  | 3,504 (607) | 9 (-78 to 96) |  | 6^a^ (-) | 1·23 (0·60-2·52) |  | 20 (13) | 1·24 (0·77-2·00) |
| Clobazam | 356 |  | 3,326 (640) | -123 (-180 to -67) |  | 33 (9) | 1·93 (1·33-2·79) |  | 57 (16) | 1·45 (1·09-1·94) |
| Primidone | 57 |  | 3,276 (706) | -220 (-360 to -80) |  | 8 (14) | 3·36 (1·56-7·24) |  | 11 (19) | 1·94 (0·99-3·81) |
| Zonisamide | 103 |  | 3,317 (616) | -114 (-220 to -7) |  | 6 (6) | 1·17 (0·51-2·70) |  | 18 (18) | 1·58 (0·94-2·66) |
| Vigabatrin | 88 |  | 3,371 (567) | -113 (-226 to 1) |  | <5 | NA |  | 16 (18) | 1·73 (0·99-3·00) |
| Ethosuximide | 52 |  | 3,499 (584) | 12 (-137 to 161) |  | <5 | NA |  | 7 (14) | 1·37 (0·61-3·09) |
| Lacosamide | 55 |  | 3,213 (757) | -221 (-366 to -76) |  | 7 (13) | 2·75 (1·22-6·20) |  | 10 (18) | 1·67 (0·83-3·37) |
| Tiagabine | 12 |  | 3,304 (725) | -163 (-468 to 141) |  | <5 | NA |  | <5 | NA |
| Eslicarbazepine | 10 |  | 3,382 (397) | 29 (-304 to 362) |  | 0 | NA |  | <5 | NA |
| Perampanel | <5 |  | NA | NA |  | NA | NA |  | NA | NA |
| Rufinamide | <5 |  | NA | NA |  | NA | NA |  | NA | NA |
| Brivaracetam | <5 |  | NA | NA |  | NA | NA |  | NA | NA |
| Sultiame | 0 |  | NA | NA |  | NA | NA |  | NA | NA |
| Felbamate | 0 |  | NA | NA |  | NA | NA |  | NA | NA |
| Retigabine | 0 |  | NA | NA |  | NA | NA |  | NA | NA |
| Aminobuturic | 0 |  | NA | NA |  | NA | NA |  | NA | NA |
| NA = Not analyzed due to low numbers.  ^a^ Number is copied from Table 2a for data privacy reasons, as differences are <5. However, analyses are based on actual numbers. | | | | | | | | | | |
| Adjustment: Country of birth, year of birth, sex of child, maternal age, parity, cohabitation, pre-pregnancy hospital admittances, maternal education, smoking in pregnancy, maternal psychiatric disorders, use of psychotropic drugs in pregnancy, and maternal epilepsy. | | | | | | | | | | |

| **Table S9.** Birth weight, low birth weight (<2,500 g), and small for gestational age (SGA) in 38,714 children of **mothers with epilepsy** exposed and unexposed to antiseizure medication (ASM) in **mono- and polytherapy** during pregnancy | | | | | | | | | | |
| --- | --- | --- | --- | --- | --- | --- | --- | --- | --- | --- |
|  | *Exposed* |  | **Birth weight**  **(grams)** | |  | **Low birth weight**  **(<2,500 g)** | |  | **Small for gestational age (SGA)** | |
| Exposure groups | *n* |  | *Mean (s.d.)* | Adjusted mean difference  (95% CI) |  | *N (%)* | Adjusted OR  (95% CI) |  | *N (%)* | Adjusted OR  (95% CI) |
| No ASM | 22,227 |  | 3,472 (583) | 0·00 (ref) |  | 1,006 (4·5) | 1·00 (ref) |  | 2,416 (10·9) | 1·00 (ref) |
| Any ASM | 16,487 |  | 3,457 (606) | -15 (-28 to -2) |  | 859 (5·2) | 1·15 (1·04-1·27) |  | 2,118 (12·8) | 1·14 (1·07-1·22) |
|  |  |  |  |  |  |  |  |  |  |  |
| **Mono- and polytherapy** |  |  |  |  |  |  |  |  |  |  |
| Lamotrigine | 6,940 |  | 3,484 (594) | 16 (0 to 32) |  | 314 (4·5) | 0·98 (0·86-1·13) |  | 766 (11·0) | 0·99 (0·91-1·09) |
| Carbamazepine | 3,316 |  | 3,426 (633) | -77 (-101 to -53) |  | 204 (6·2) | 1·49 (1·24-1·79) |  | 465 (14·0) | 1·39 (1·23-1·58) |
| Valproate | 2,779 |  | 3,493 (628) | 19 (-7 to 45) |  | 149 (5·4) | 1·18 (0·96-1·45) |  | 355 (12·8) | 1·14 (0·99-1·31) |
| Pregabalin | 220 |  | 3,273 (623) | -84 (-162 to -6) |  | 17 (8) | 1·01 (0·59-1·72) |  | 34 (16) | 1·28 (0·87-1·88) |
| Oxcarbazepine | 1,993 |  | 3,397 (605) | -83 (-114 to -52) |  | 119 (6·0) | 1·41 (1·11-1·79) |  | 363 (18·2) | 1·75 (1·50-2·04) |
| Clonazepam | 816 |  | 3,341 (631) | -77 (-119 to -36) |  | 71 (9) | 1·51 (1·15-1·98) |  | 136 (16·7) | 1·42 (1·16-1·74) |
| Gabapentin | 238 |  | 3,359 (530) | -62 (-135 to 12) |  | 8^a^ (-) | 0·85 (0·47-1·54) |  | 33 (14) | 1·18 (0·81-1·74) |
| Levetiracetam | 2,082 |  | 3,428 (571) | -50 (-77 to -23) |  | 99^b^ (-) | 1·07 (0·86-1·34) |  | 285 (13·7) | 1·30 (1·13-1·49) |
| Topiramate | 665 |  | 3,338 (599) | -115 (-160 to -71) |  | 51 (8) | 1·61 (1·19-2·17) |  | 139 (20·9) | 2·04 (1·67-2·49) |
| Phenobarbital | 87 |  | 3,387 (540) | -64 (-184 to 57) |  | <5 | NA |  | 7^a^ (-) | 1·08 (0·57-2·07) |
| Acetazolamide | 9^a^ |  | 3,295 (567) | -64 (-400 to 272) |  | <5 | NA |  | <5 | NA |
| Phenytoin | 129 |  | 3,458 (604) | -8 (-109 to 92) |  | 6^a^ (-) | 1·17 (0·54-2·55) |  | 18 (14) | 1·23 (0·73-2·06) |
| Clobazam | 335 |  | 3,320 (634) | -113 (-175 to -51) |  | 33^b^ (-) | 1·81 (1·23-2·67) |  | 57^b^ (-) | 1·41 (1·04-1·92) |
| Primidone | 47 |  | 3,235 (719) | -225 (-389 to -61) |  | 8^b^ (-) | 2·58 (1·06-6·25) |  | 11 (23) | 2·28 (1·12-4·62) |
| Zonisamide | 103 |  | 3,317 (616) | -122 (-235 to -10) |  | 6 (6) | 1·23 (0·53-2·85) |  | 18 (18) | 1·68 (0·99-2·84) |
| Vigabatrin | 75 |  | 3,373 (581) | -78 (-209 to 53) |  | <5 | NA |  | 16^b^ (-) | 1·49 (0·81-2·74) |
| Ethosuximide | 52^b^ |  | 3,505 (595) | 17 (-145 to 178) |  | <5 | NA |  | 7 (14) | 1·41 (0·62-3·21) |
| Lacosamide | 55 |  | 3,213 (757) | -222 (-376 to -68) |  | 7 (13) | 2·84 (1·25-6·43) |  | 10 (18) | 1·80 (0·89-3·66) |
| Tiagabine | 12^b^ |  | 3,497 (548) | 31 (-322 to 384) |  | <5 | NA |  | <5 | NA |
| Eslicarbazepine | 10 |  | 3,382 (397) | 27 (-325 to 379) |  | <5 | NA |  | <5 | NA |
| Perampanel | <5 |  | NA | NA |  | NA | NA |  | NA | NA |
| Rufinamide | <5 |  | NA | NA |  | NA | NA |  | NA | NA |
| Brivaracetam | <5 |  | NA | NA |  | NA | NA |  | NA | NA |
| Sultiame | 0 |  | NA | NA |  | NA | NA |  | NA | NA |
| Felbamate | 0 |  | NA | NA |  | NA | NA |  | NA | NA |
| Retigabine | 0 |  | NA | NA |  | NA | NA |  | NA | NA |
| Aminobuturic | 0 |  | NA | NA |  | NA | NA |  | NA | NA |
| NA = Not analyzed due to low numbers.  ^a^ Number is copied from Table 2b for data privacy reasons, as differences are <5. However, analyses are based on actual numbers.  ^b^ Number is copied from sTable 8 for data privacy reasons, as differences are <5. However, analyses are based on actual numbers. | | | | | | | | | | |
| Adjustment: Country of birth, year of birth, sex of child, maternal age, parity, cohabitation, pre-pregnancy hospital admittances, maternal education, smoking in pregnancy, maternal psychiatric disorders, use of psychotropic drugs in pregnancy. | | | | | | | | | | |

| **Table S10.** Head circumference and microcephaly in 4,494,918 children exposed and unexposed to antiseizure medication (ASM) in **mono- and polytherapy** during pregnancy | | | | | | | |
| --- | --- | --- | --- | --- | --- | --- | --- |
|  | *Exposed* |  | **Head circumference, cm** | |  | **Microcephaly** | |
| Exposure groups | *n* |  | *Mean (s.d.)* | Adjusted mean difference (cm)  (95 % CI) |  | *N* (%)* | Adjusted OR  (95 % CI) |
| No ASM | 4,467,848 |  | 35·0 (1·8) | 0·00 (ref) |  | 134,023 (3·0) | 1·00 (ref) |
| Any ASM | 27,070 |  | 34·8 (1·9) | -0·06 (-0·08 to -0·04) |  | 992 (3·7) | 1·09 (1·01-1·18) |
|  |  |  |  |  |  |  |  |
| **Mono- and polytherapy** |  |  |  |  |  |  |  |
| Lamotrigine | 10,609 |  | 34·8 (1·9) | 0·04 (0·00 to 0·07) |  | 346 (3·3) | 0·98 (0·87-1·11) |
| Carbamazepine | 4,171 |  | 34·7 (2·1) | -0·30 (-0·35 to -0·24) |  | 177 (4·2) | 1·50 (1·25-1·79) |
| Valproate | 3,638 |  | 34·8 (1·9) | -0·02 (-0·08 to 0·03) |  | 148 (4·1) | 1·22 (1·00-1·48) |
| Pregabalin | 2,586 |  | 34·6 (1·8) | -0·10 (-0·16 to -0·03) |  | 107 (4·1) | 1·10 (0·90-1·35) |
| Oxcarbazepine | 2,145 |  | 34·8 (1·9) | -0·16 (-0·24 to -0·08) |  | 86 (4·0) | 1·27 (0·99-1·62) |
| Clonazepam | 2,039 |  | 34·6 (1·9) | -0·19 (-0·27 to -0·11) |  | 95 (4·7) | 1·23 (0·98-1·53) |
| Gabapentin | 1,556 |  | 34·8 (1·8) | -0·11 (-0·19 to -0·02) |  | 43 (2·8) | 0·78 (0·56-1·08) |
| Levetiracetam | 2,098 |  | 34·7 (1·8) | -0·13 (-0·20 to -0·05) |  | 86 (4·1) | 1·28 (1·01-1·62) |
| Topiramate | 1,045 |  | 34·8 (1·8) | -0·07 (-0·18 to 0·03) |  | 47 (4·5) | 1·34 (0·99-1·82) |
| Phenobarbital | 228 |  | 34·6 (1·8) | -0·37 (-0·59 to -0·15) |  | 11 (5·0) | 1·39 (0·74-2·64) |
| Acetazolamide | 127^a^ |  | 35·0 (1·5) | 0·13 (-0·16 to 0·42) |  | <5 | NA |
| Phenytoin | 152 |  | 35·0 (2·0) | 0·02 (-0·27 to 0·31) |  | 5 (3·0) | 1·01 (0·36-2·83) |
| Clobazam | 356 |  | 34·5 (2·0) | -0·35 (-0·53 to -0·17) |  | 22 (6·2) | 1·82 (1·16-2·88) |
| Primidone | 57 |  | 34·1 (2·4) | -0·88 (-1·33 to -0·44) |  | 5 (9·0) | 3·17 (1·24-8·07) |
| Zonisamide | 103 |  | 34·3 (2·1) | -0·44 (-0·76 to -0·11) |  | 12 (11·7) | 3·66 (1·98-6·76) |
| Vigabatrin | 88 |  | 35·1 (1·8) | 0·15 (-0·21 to 0·51) |  | 6 (6·3) | 2·05 (0·82-5·12) |
| Ethosuximide | 52 |  | 34·6 (1·8) | -0·31 (-0·79 to 0·17) |  | <5 | NA |
| Lacosamide | 55 |  | 34·1 (2·1) | -0·69 (-1·13 to -0·24) |  | <5 | NA |
| Tiagabine | 12 |  | 34·5 (2·7) | -0·40 (-1·40 to 0·60) |  | <5 | NA |
| Eslicarbazepine | 10 |  | 35·1 (1·4) | 0·46 (-0·56 to 1·48) |  | 0 | NA |
| Perampanel | <5 |  | NA | NA |  | NA | NA |
| Rufinamide | <5 |  | NA | NA |  | NA | NA |
| Brivaracetam | <5 |  | NA | NA |  | NA | NA |
| Sultiame | 0 |  | NA | NA |  | NA | NA |
| Felbamate | 0 |  | NA | NA |  | NA | NA |
| Retigabine | 0 |  | NA | NA |  | NA | NA |
| Aminobuturic | 0 |  | NA | NA |  | NA | NA |
| Adjustment: Country of birth, year of birth, sex of child, maternal age, parity, cohabitation, pre-pregnancy hospital admittances, maternal education, smoking in pregnancy, maternal psychiatric disorders, maternal epilepsy, use of psychotropic drugs in pregnancy, and gestational age at birth.  *Rounded average of 20 imputed Ns. | | | | | | | |

| **Table S11.** Head circumference and microcephaly in 38,714 children of **mothers with epilepsy** exposed and unexposed to antiseizure medication (ASM) in **mono- and polytherapy** during pregnancy | | | | | | | |
| --- | --- | --- | --- | --- | --- | --- | --- |
|  | *Exposed* |  | **Head circumference, cm** | |  | **Microcephaly** | |
| Exposure groups | *n* |  | *Mean (s.d.)* | Adjusted mean difference (cm) |  | *N* (%)* | Adjusted OR |
| No ASM | 22,227 |  | 34·9 (1·8) | 0·00 (ref) |  | 699 (3·1) | 1·00 (ref) |
| Any ASM | 16,487 |  | 34·8 (1·9) | -0·06 (-0·09 to -0·02) |  | 588 (3·6) | 1·09 (0·96-1·23) |
|  |  |  |  |  |  |  |  |
| **Mono- and polytherapy** |  |  |  |  |  |  |  |
| Lamotrigine | 6,940 |  | 34·9 (1·9) | 0·04 (-0·01 to 0·09) |  | 211 (3·0) | 0·95 (0·80-1·11) |
| Carbamazepine | 3,316 |  | 34·7 (2·1) | -0·35 (-0·43 to -0·28) |  | 143 (4·3) | 1·64 (1·31-2·06) |
| Valproate | 2,779 |  | 34·9 (2·0) | -0·02 (-0·11 to 0·06) |  | 115 (4·1) | 1·29 (1·00-1·66) |
| Pregabalin | 220 |  | 34·3 (2·1) | -0·25 (-0·49 to -0·00) |  | 11 (5·1) | 1·42 (0·75-2·68) |
| Oxcarbazepine | 1,993 |  | 34·7 (1·9) | -0·14 (-0·24 to -0·03) |  | 82 (4·1) | 1·22 (0·90-1·65) |
| Clonazepam | 816 |  | 34·6 (2·1) | -0·19 (-0·32 to -0·06) |  | 29 (3·5) | 1·02 (0·67-1·57) |
| Gabapentin | 238 |  | 34·7 (1·7) | -0·09 (-0·32 to 0·13) |  | 5 (2·0) | 0·59 (0·22-1·58) |
| Levetiracetam | 2,082 |  | 34·7 (1·8) | -0·12 (-0·20 to -0·04) |  | 85 (4·1) | 1·24 (0·97-1·59) |
| Topiramate | 665 |  | 34·7 (1·8) | -0·12 (-0·26 to 0·02) |  | 29 (4·3) | 1·32 (0·89-1·98) |
| Phenobarbital | 87 |  | 34·6 (1·5) | -0·31 (-0·68 to 0·07) |  | <5 | NA |
| Acetazolamide | 9^a^ |  | 34·6 (1·8) | -0·01 (-1·05 to 1·03) |  | 0 | NA |
| Phenytoin | 129 |  | 34·9 (1·9) | -0·04 (-0·37 to 0·29) |  | <5 | NA |
| Clobazam | 335 |  | 34·4 (2·0) | -0·33 (-0·53 to -0·13) |  | 22 (6·6) | 1·83 (1·15-2·92) |
| Primidone | 47 |  | 34·0 (2·5) | -0·96 (-1·49 to -0·44) |  | 5 (11·0) | 3·86 (1·46-10·21) |
| Zonisamide | 103 |  | 34·3 (2·1) | -0·46 (-0·81 to -0·11) |  | 12 (11·7) | 3·80 (2·04-7·10) |
| Vigabatrin | 75 |  | 35·2 (1·9) | 0·20 (-0·21 to 0·61) |  | <5 | NA |
| Ethosuximide | 52^b^ |  | 34·7 (1·8) | -0·30 (-0·82 to 0·22) |  | <5 | NA |
| Lacosamide | 55 |  | 34·1 (2·1) | -0·67 (-1·15 to -0·19) |  | <5 | NA |
| Tiagabine | 12^b^ |  | 34·9 (2·5) | 0·05 (-1·12 to 1·22) |  | <5 | NA |
| Eslicarbazepine | 10 |  | 35·1 (1·4) | 0·46 (-0·63 to 1·56) |  | 0 | NA |
| Perampanel | <5 |  | NA | NA |  | NA | NA |
| Rufinamide | <5 |  | NA | NA |  | NA | NA |
| Brivaracetam | <5 |  | NA | NA |  | NA | NA |
| Sultiame | 0 |  | NA | NA |  | NA | NA |
| Felbamate | 0 |  | NA | NA |  | NA | NA |
| Retigabine | 0 |  | NA | NA |  | NA | NA |
| Aminobuturic | 0 |  | NA | NA |  | NA | NA |
| NA = Not analyzed due to low numbers. | | | | | | | |
| ^a^ Number is copied from Table 2b for data privacy reasons, as differences are <5. However, analyses are based on actual numbers.  ^b^ Number is copied from sTable 8 for data privacy reasons, as differences are <5. However, analyses are based on actual numbers.  Adjustment: Country of birth, year of birth, sex of child, maternal age, parity, cohabitation, pre-pregnancy hospital admittances, maternal education, smoking in pregnancy, maternal psychiatric disorders, and use of psychotropic drugs in pregnancy. | | | | | | | |
| *Rounded average of 20 imputed Ns. | | | | | | | |

| **Table S12.** Small for gestational age and microcephaly in 25,176 children of mothers with “**active**”****** epilepsy exposed and unexposed to antiseizure medication (ASM) in monotherapy during pregnancy | | | | | | | |
| --- | --- | --- | --- | --- | --- | --- | --- |
|  | *Exposed* |  | **Small for gestational age** | |  | **Microcephaly** | |
| Exposure groups | *n* |  | *N (%)* | Adjusted OR |  | *N* (%)* | Adjusted OR |
| No ASM | 9,770 |  | 1,115 (11.4) | 1·00 (ref.) |  | 296 (3·0) | 1·00 (ref.) |
| Any ASM | 15,406 |  | 1,974 (12.8) | 1·09 (1·00-1·19) |  | 553 (3·6) | 1·12 (0·95-1·31) |
|  |  |  |  |  |  |  |  |
| **Monotherapies** |  |  |  |  |  |  |  |
| Lamotrigine | 4,836 |  | 497 (10.3) | 0·91 (0·81-1·03) |  | 137 (2·8) | 0·89 (0·71-1·11) |
| Carbamazepine | 2,565 |  | 329 (12.8) | 1·22 (1·05-1·42) |  | 101 (4·0) | 1·38 (1·05-1·82) |
| Valproate | 1,852 |  | 213 (11.5) | 0·99 (0·82-1·19) |  | 63 (3·4) | 0·98 (0·70-1·37) |
| Pregabalin | 56 |  | 9 (16) | 1·24 (0·59-2·61) |  | <5 | NA |
| Oxcarbazepine | 1,390 |  | 207 (14.9) | 1·30 (1·05-1·59) |  | 46 (3·3) | 0·89 (0·59-1·33) |
| Clonazepam | 274 |  | 45 (16) | 1·31 (0·92-1·86) |  | 7 (2·6) | 0·68 (0·30-1·56) |
| Gabapentin | 96 |  | 15^a^ (-) | 0·93 (0·49-1·77) |  | <5 | NA |
| Levetiracetam | 1,044 |  | 125^a^ (-) | 1·02 (0·82-1·27) |  | 29 (2·7) | 0·78 (0·51-1·19) |
| Topiramate | 260 |  | 48 (19) | 1·83 (1·31-2·55) |  | 10 (4·0) | 1·26 (0·65-2·42) |
| Phenobarbital | 47^a^ |  | 7^a^ (-) | 1·07 (0·41-2·78) |  | <5 | NA |
| Acetazolamide | <5 |  | <5 | NA |  | <5 | NA |
| Phenytoin | 64^a^ |  | 7^a^ (-) | 0·60 (0·24-1·54) |  | <5 | NA |
| Clobazam | 27^a^ |  | <5 | NA |  | <5 | NA |
| Primidone | 22 |  | 6^a^ (-) | 1·99 (0·69-5·71) |  | <5 | NA |
| Zonisamide | 19 |  | <5 | NA |  | <5 | NA |
| Vigabatrin | 9^a^ |  | <5 | NA |  | <5 | NA |
| Ethosuximide | 11 |  | <5 | NA |  | <5 | NA |
| Lacosamide | 9 |  | <5 | NA |  | <5 | NA |
| NA = Not analyzed due to low numbers. | | | | | | | |
| ^a^ Number is copied from Table 2b for data privacy reasons, as differences are <5. However, analyses are based on actual numbers.  Adjustment: Country of birth, year of birth, sex of child, maternal age, parity, cohabitation, pre-pregnancy hospital admittances, maternal education, smoking in pregnancy, maternal psychiatric disorders, and use of psychotropic drugs in pregnancy. | | | | | | | |
| *Rounded average of 20 imputed Ns.  **Active epilepsy is defined as maternal diagnosis of epilepsy or use of antiseizure medication within one year of birth. | | | | | | | |

| **Table S13.** Complete case analyses of small for gestational age and microcephaly in 3,499,545 children exposed and unexposed to antiseizure medication (ASM) in monotherapy during pregnancy | | | | | | | |  |
| --- | --- | --- | --- | --- | --- | --- | --- | --- |
|  | *Exposed* |  | **Small for gestational age** | |  | **Microcephaly** | |  |
| Exposure groups | *n* |  | *N (%)* | Adjusted OR |  | *N* (%)* | Adjusted OR |  |
| No ASM | 3,478,122 |  | 339,246 (9·8) | 1·00 (ref.) |  | 101,850 (2·9) | 1·00 (ref.) |  |
| Any ASM | 21,423 |  | 2,684 (12·5) | 1·10 (1·05-1·16) |  | 780 (3·6) | 1·07 (0·99-1·17) |  |
|  |  |  |  |  |  |  |  |  |
| **Monotherapies** |  |  |  |  |  |  |  |  |
| Lamotrigine | 7,766 |  | 804 (10·4) | 0·91 (0·84-0·99) |  | 238 (3·1) | 0·92 (0·80-1·05) |  |
| Carbamazepine | 2,128 |  | 262 (12·3) | 1·27 (1·10-1·45) |  | 90 (4) | 1·58 (1·26-1·97) |  |
| Valproate | 1,819 |  | 225 (12·4) | 1·10 (0·95-1·28) |  | 69 (4) | 1·14 (0·88-1·47) |  |
| Pregabalin | 1,965 |  | 276 (14·0) | 1·12 (0·98-1·27) |  | 72 (4) | 0·97 (0·76-1·24) |  |
| Oxcarbazepine | 1,124 |  | 161 (14·3) | 1·37 (1·15-1·64) |  | 35 (3) | 0·99 (0·70-1·40) |  |
| Clonazepam | 959 |  | 158 (16·5) | 1·26 (1·05-1·50) |  | 45 (5) | 1·18 (0·87-1·61) |  |
| Gabapentin | 1,081 |  | 131 (12·1) | 1·09 (0·91-1·32) |  | 27 (3) | 0·69 (0·47-1·03) |  |
| Levetiracetam | 941 |  | 110 (11·7) | 1·10 (0·89-1·35) |  | 28 (3) | 0·90 (0·61-1·33) |  |
| Topiramate | 572 |  | 89 (16) | 1·55 (1·23-1·95) |  | 26 (5) | 1·44 (0·97-2·14) |  |
| Phenobarbital | 101 |  | 12 (12) | 1·03 (0·56-1·91) |  | 6 (6) | 1·61 (0·65-3·98) |  |
| Acetazolamide | 116 |  | 15 (13) | 1·13 (0·66-1·96) |  | <5 | NA |  |
| Phenytoin | 37 |  | <5 | NA |  | <5 | NA |  |
| Clobazam | 31 |  | <5 | NA |  | <5 | NA |  |
| Primidone | 27 |  | 6^a^ (-) | 2·03 (0·75-5·51) |  | <5 | NA |  |
| Zonisamide | 19^a^ |  | <5 | NA |  | <5 | NA |  |
| Vigabatrin | 10 |  | <5 | NA |  | <5 | NA |  |
| Ethosuximide | 11^a^ |  | <5 | NA |  | <5 | NA |  |
| Lacosamide | 9^a^ |  | <5 | NA |  | <5 | NA |  |
| NA = Not analyzed due to low numbers. | | | | | | | |  |
| ^a^ Number is copied from Table 2a for data privacy reasons, as differences are <5. However, analyses are based on actual numbers.  Adjustment: Country of birth, year of birth, sex of child, maternal age, parity, cohabitation, pre-pregnancy hospital admittances, maternal education, smoking in pregnancy, maternal psychiatric disorders, and use of psychotropic drugs in pregnancy. | | | | | | | |  |
| *Rounded average of 20 imputed Ns. | | | | | | | |  |
| **Table S14.** Small for gestational age and microcephaly in 4,494,918 children exposed and unexposed to antiseizure medication (ASM) in monotherapy during pregnancy after restricting the exposure period to the period from LMP** to birth. | | | | | | | | |
|  | *Exposed* |  | **Small for gestational age** | |  | **Microcephaly** | | |
| Exposure groups | *n* |  | *N (%)* | Adjusted OR |  | *N* (%)* | Adjusted OR | |
| No ASM | 4,470,101 |  | 446,537 (10·0) | 1·00 (ref.) |  | 134,099 (3·0) | 1·00 (ref.) | |
| Any ASM | 24,817 |  | 3,209 (12·9) | 1·14 (1·09-1·20) |  | 916 (3·7) | 1·11 (1·02-1·20) | |
|  |  |  |  |  |  |  |  | |
| **Monotherapies** |  |  |  |  |  |  |  | |
| Lamotrigine | 8,109 |  | 850 (10·5) | 0·92 (0·85-0·99) |  | 261 (3·2) | 0·97 (0·85-1·11) | |
| Carbamazepine | 3,294 |  | 409 (12·4) | 1·25 (1·12-1·40) |  | 127 (3·9) | 1·41 (1·15-1·74) | |
| Valproate | 2,456 |  | 297 (12·1) | 1·07 (0·94-1·22) |  | 90 (3·7) | 1·11 (0·86-1·42) | |
| Pregabalin | 1,713 |  | 258 (15·1) | 1·22 (1·06-1·40) |  | 66 (3·9) | 1·03 (0·79-1·33) | |
| Oxcarbazepine | 1,561 |  | 241^a^ (-) | 1·49 (1·29-1·72) |  | 51 (3·3) | 1·05 (0·77-1·43) | |
| Clonazepam | 1,215 |  | 205 (16·9) | 1·32 (1·13-1·54) |  | 54 (4·4) | 1·12 (0·84-1·50) | |
| Gabapentin | 989 |  | 117 (11·8) | 1·04 (0·86-1·27) |  | 21 (2·1) | 0·58 (0·37-0·93) | |
| Levetiracetam | 1,066 |  | 125^a^ (-) | 1·09 (0·89-1·32) |  | 32 (3·0) | 0·91 (0·62-1·33) | |
| Topiramate | 489 |  | 83 (17) | 1·69 (1·33-2·16) |  | 20 (4·0) | 1·25 (0·79-1·98) | |
| Phenobarbital | 177 |  | 29^a^ (-) | 1·47 (0·97-2·22) |  | 11 (6·5) | 1·90 (1·00-3·63) | |
| Acetazolamide | 98 |  | 15^a^ (-) | 1·25 (0·70-2·21) |  | <5 | NA | |
| Phenytoin | 81^a^ |  | 7 (9) | 0·81 (0·37-1·78) |  | <5 | NA | |
| Clobazam | 44^a^ |  | 5 (12) | 1·22 (0·47-3·16) |  | <5 | NA | |
| Primidone | 34^a^ |  | 6 (19) | 2·06 (0·82-5·17) |  | <5 | NA | |
| Zonisamide | 19 |  | <5 | NA |  | <5 | NA | |
| Vigabatrin | 17^a^ |  | <5 | NA |  | <5 | NA | |
| Ethosuximide | 11^a^ |  | <5 | NA |  | <5 | NA | |
| Lacosamide | 9 |  | <5 | NA |  | <5 | NA | |
| NA = Not analyzed due to low numbers. | | | | | | | | |
| ^a^ Number is copied from Table 2a for data privacy reasons, as differences are <5. However, analyses are based on actual numbers.  Adjustment: Country of birth, year of birth, sex of child, maternal age, parity, cohabitation, pre-pregnancy hospital admittances, maternal education, smoking in pregnancy, maternal psychiatric disorders, and use of psychotropic drugs in pregnancy. | | | | | | | | |
| *Rounded average of 20 imputed Ns. **LMP: First day of the last menstrual period. | | | | | | | | |

| **Table S15.** Head circumference and microcephaly in 4,299,375 children exposed and unexposed to antiseizure medication (ASM) after excluding 195,543 children with congenital malformations. | | | | | | | |
| --- | --- | --- | --- | --- | --- | --- | --- |
|  | *Exposed* |  | **Head circumference, cm** | |  | **Microcephaly** | |
| Exposure groups | *n* |  | *Mean (s.d.)* | Adjusted mean difference (cm)  (95 % CI) |  | *N* (%)* | Adjusted OR  (95 % CI) |
| No ASM | 4,273,996 |  | 35·0 (1·8) | 1·00 (ref) |  | 126,370 (3·0) | 1·00 (ref) |
| Any ASM | 25,379 |  | 34·8 (1·9) | -0·06 (-0·08 to -0·03) |  | 905 (3·6) | 1·08 (1·00-1·17) |
|  |  |  |  |  |  |  |  |
| **Mono- and polytherapy** |  |  |  |  |  |  |  |
| Lamotrigine | 8,262 |  | 34·9 (1·8) | 0·06 (0·02 to 0·10) |  | 254 (3·1) | 0·95 (0·83-1·08) |
| Carbamazepine | 3,271 |  | 34·7 (2·1) | -0·27 (-0·33 to -0·20) |  | 128 (3·9) | 1·46 (1·19-1·79) |
| Valproate | 2,434 |  | 34·9 (1·8) | 0·06 (-0·01 to 0·13) |  | 86 (3·5) | 1·06 (0·82-1·37) |
| Pregabalin | 2,079 |  | 34·6 (1·7) | -0·10 (-0·17 to -0·03) |  | 75 (3·6) | 0·98 (0·77-1·25) |
| Oxcarbazepine | 1,508 |  | 34·9 (1·8) | -0·02 (-0·11 to 0·07) |  | 47 (3·1) | 1·00 (0·72-1·38) |
| Clonazepam | 1,274 |  | 34·6 (1·9) | -0·15 (-0·25 to -0·06) |  | 56 (4·4) | 1·16 (0·88-1·55) |
| Gabapentin | 1,251 |  | 34·8 (1·8) | -0·10 (-0·19 to -0·01) |  | 31 (2·5) | 0·69 (0·47-1·01) |
| Levetiracetam | 1,030 |  | 34·9 (1·8) | 0·02 (-0·08 to 0·13) |  | 31 (3·0) | 0·93 (0·63-1·36) |
| Topiramate | 585 |  | 34·9 (1·7) | -0·03 (-0·16 to 0·10) |  | 22 (3·8) | 1·22 (0·79-1·87) |
| Phenobarbital | 170 |  | 34·6 (1·8) | -0·41 (-0·66 to -0·16) |  | 9 (5·5) | 1·61 (0·79-3·29) |
| Acetazolamide | 117 |  | 35·0 (1·4) | 0·15 (-0·15 to 0·44) |  | <5 | NA |
| Phenytoin | 75 |  | 35·3 (1·9) | 0·21 (-0·21 to 0·62) |  | <5 | NA |
| Clobazam | 44^a^ |  | 35·0 (2·3) | -0·02 (-0·53 to 0·49) |  | <5 | NA |
| Primidone | 34^a^ |  | 34·6 (1·4) | -0·44 (-1·01 to 0·13) |  | <5 | NA |
| Zonisamide | 19^a^ |  | 34·0 (1·8) | -0·74 (-1·54 to 0·05) |  | <5 | NA |
| Vigabatrin | 17 |  | 35·7 (1·7) | 0·81 (0·02 to 1·59) |  | <5 | NA |
| Ethosuximide | 11^a^ |  | 34·3 (2·6) | -0·42 (-1·43 to 0·59) |  | <5 | NA |
| Lacosamide | 9 |  | 35·4 (1·4) | 0·77 (-0·35 to 1·90) |  | <5 | NA |
| ^a^ Number is copied from Table 2a for data privacy reasons, as differences are <5. However, analyses are based on actual numbers.  Adjustment: Country of birth, year of birth, sex of child, maternal age, parity, cohabitation, pre-pregnancy hospital admittances, maternal education, smoking in pregnancy, maternal psychiatric disorders, maternal epilepsy, use of psychotropic drugs in pregnancy, and gestational age at birth. | | | | | | | |

| **Table S16.** Small for gestational age and microcephaly for children from the overall population and in children of mothers with epilepsy exposed and unexposed to carbamazepine and lamotrigine in monotherapy during pregnancy. | | | | | | |
| --- | --- | --- | --- | --- | --- | --- |
|  | *Exposed* | **Small for gestational age** | |  | **Microcephaly** | |
| Exposure groups | *n* | *N (%)* | Adjusted OR |  | *N* (%)* | Adjusted OR |
| All children | | | | | | |
| **Monotherapies** |  |  |  |  |  |  |
| Lamotrigine | 8,756 | 920 (10·5) | 1·00 (ref) |  | 274 (3·1) | 1·00 (ref) |
| Carbamazepine | 3,424 | 428 (12·5) | 1·37 (1·17-1·60) |  | 135 (3·9) | 1·78 (1·36-2·32) |
| Children of mothers with epilepsy | | | | | | |
| **Monotherapies** |  |  |  |  |  |  |
| Lamotrigine | 5,299 | 546 (10·3) | 1·00 (ref) |  | 151 (2·8) | 1·00 (ref) |
| Carbamazepine | 2,669 | 341 (12·8) | 1·39 (1·16-1·65) |  | 105 (3·9) | 1·70 (1·25-2·31) |
|  |  |  |  |  |  |  |
| Adjustment: Country of birth, year of birth, sex of child, maternal age, parity, cohabitation, pre-pregnancy hospital admittances, maternal education, smoking in pregnancy, maternal psychiatric disorders, maternal epilepsy, and use of psychotropic drugs in pregnancy. | | | | | | |
| * Rounded average of imputed N’s | |  |  |  |  |  |

| **Table S17.** Small for gestational age and microcephaly in 3,041,503 children with information of maternal body mass index (BMI) exposed and unexposed to antiseizure medication (ASM). | | | | | | | | | |
| --- | --- | --- | --- | --- | --- | --- | --- | --- | --- |
|  | *Exposed* |  | **Small for gestational age** | | |  | **Microcephaly** | | |
| Exposure groups | *n* |  | *N* (%)* | Adjusted OR  without BMI**  (95 % CI) | Adjusted OR with BMI**  (95 % CI) |  | *N* (%)* | Adjusted OR without BMI**  (95 % CI) | Adjusted OR with BMI**  (95 % CI) |
| No ASM | 3,021,897 |  | 301,445 (10·0) | 1·00 (ref) | 1·00 (ref) |  | 92,338 (3·1) | 1·00 (ref) | 1·00 (ref) |
| Any ASM | 19,606 |  | 2,497 (12·7) | 1·12 (1·07-1·18) | 1·13 (1·08-1·19) |  | 748 (3·8) | 1·10 (1·01-1·20) | 1·11 (1·02-1·21) |
|  |  |  |  |  |  |  |  |  |  |
| **Monotherapies** |  |  |  |  |  |  |  |  |  |
| Lamotrigine | 7,137 |  | 752 (10·5) | 0·93 (0·86-1·01) | 0·94 (0·87-1·03) |  | 238 (3·3) | 0·98 (0·85-1·13) | 0·99 (0·86-1·14) |
| Carbamazepine | 1,828 |  | 235 (12·9) | 1·32 (1·14-1·52) | 1·32 (1·14-1·53) |  | 82 (4·5) | 1·66 (1·31-2·11) | 1·66 (1·31-2·11) |
| Valproate | 1,636 |  | 205 (12·5) | 1·11 (0·95-1·29) | 1·12 (0·96-1·30) |  | 66 (4·0) | 1·17 (0·90-1·53) | 1·18 (0·91-1·54) |
| Pregabalin | 1,958 |  | 288 (14·7) | 1·19 (1·05-1·35) | 1·21 (1·06-1·37) |  | 74 (3·8) | 0·98 (0·77-1·25) | 0·99 (0·78-1·26) |
| Oxcarbazepine | 1,006 |  | 137 (13·6) | 1·28 (1·06-1·54) | 1·30 (1·08-1·57) |  | 32 (3·1) | 0·97 (0·67-1·40) | 0·98 (0·68-1·42) |
| Clonazepam | 825 |  | 145 (17·6) | 1·33 (1·10-1·60) | 1·25 (1·03-1·50) |  | 45 (5·4) | 1·29 (0·94-1·76) | 1·23 (0·90-1·68) |
| Gabapentin | 1,096 |  | 136 (12·4) | 1·13 (0·94-1·36) | 1·15 (0·96-1·38) |  | 21 (2·0) | 0·52 (0·33-0·82) | 0·53 (0·34-0·84) |
| Levetiracetam | 908 |  | 108 (11·9) | 1·10 (0·89-1·36) | 1·09 (0·88-1·34) |  | 28 (3·1) | 0·92 (0·62-1·38) | 0·91 (0·61-1·37) |
| Topiramate | 533 |  | 77 (14) | 1·40 (1·10-1·80) | 1·47 (1·15-1·89) |  | 24 (4·5) | 1·40 (0·93-2·12) | 1·46 (0·97-2·21) |
| Phenobarbital | 54 |  | 7 (13) | 0·98 (0·44-2·21) | 0·87 (0·38-1·98) |  | 6 (11·1) | 2·97 (1·25-7·03) | 2·73 (1·15-6·50) |
| Acetazolamide | 99 |  | 15^a^ (-) | 1·06 (0·58-1·95) | 1·20 (0·65-2·21) |  | <5 | NA | NA |
| Phenytoin | 35 |  | <5 | NA | NA |  | <5 | NA | NA |
| Clobazam | 14 |  | <5 | NA | NA |  | <5 | NA | NA |
| Primidone | 14 |  | <5 | NA | NA |  | <5 | NA | NA |
| Zonisamide | 19^a^ |  | <5 | NA | NA |  | <5 | NA | NA |
| Vigabatrin | <5 |  | <5 | NA | NA |  | <5 | NA | NA |
| Ethosuximide | 11^a^ |  | <5 | NA | NA |  | <5 | NA | NA |
| Lacosamide | 9^a^ |  | <5 | NA | NA |  | <5 | NA | NA |
| * Rounded average of imputed N’s  ** OR adjusted and not adjusted for maternal body mass index (BMI)  ^a^ Number is copied from Table 2a for data privacy reasons, as differences are <5. However, analyses are based on actual numbers.  NA = Not analyzed due to low numbers or insufficient follow-up time  Adjustment: Country of birth, year of birth, sex of child, maternal age, parity, cohabitation, pre-pregnancy hospital admittances, maternal education, smoking in pregnancy, maternal psychiatric disorders, maternal epilepsy, and use of psychotropic drugs in pregnancy. | | | | | | | | | |

# **References**

1. Lupattelli A, Wood ME, Nordeng H. Analyzing Missing Data in Perinatal Pharmacoepidemiology Research: Methodological Considerations to Limit the Risk of Bias. Clin Ther 2019;41(12):2477-2487.

2. Graham JW, Olchowski AE, Gilreath TD. How many imputations are really needed? Some practical clarifications of multiple imputation theory. Prev Sci 2007;8(3):206-13.

3. Cohen JM, Alvestad S, Cesta CE, et al. Comparative Safety of Antiseizure Medication Monotherapy for Major Malformations. Ann Neurol 2023;93(3):551-562.
