# Supplementary material for: Prenatal exposure to antiseizure medications and fetal growth: a population-based cohort study from the Nordic countries
Source: Lancet Reg Health Eur. 2024 Feb 8;38:100849. doi: 10.1016/j.lanepe.2024.100849 (PMC10928302; doi:10.1016/j.lanepe.2024.100849)
Supplement: Translated Abstracts [file mmc2.docx]

# **Translated Abstract - Danish Abstract**

The translations in Norwegian, Swedish, Icelandic, Finnish and Danish were submitted by the authors, and we reproduce them as supplied. The translated abstracts have not been peer reviewed. Our editorial processes have only been applied to the original abstract in English, which should serve as reference for this manuscript.

**Resume**

**Baggrund: Lav fødselsvægt kan have både kort- og langsigtede konsekvenser, og det er usikkert hvordan moderens brug af forskellige former for epilepsimedicin under graviditeten påvirker fosterets vækst.**

**Metode**:  **Dette studie er en undersøgelse af børn født i Danmark, Finland, Island, Norge og Sverige fra 1996 til 2017. Moderens brug af epilepsimedicin under graviditeten blev identificeret fra nationale receptregistre ud fra moderens indløsning af recepter på epilepsimedicin under graviditeten. Det primære resultat i undersøgelsen var risikoen (odds ratioen (OR)) for blive født med lav fødselsvægt eller med lille hovedomfang.**

**Resultater:** I studiet indgår 4.494.920 børn (drenge: 51,3%), herunder 38.710 (0,9%) børn født af mødre med epilepsi. I den samlede gruppe af børn var moderens brug i graviditeten af carbamazepin (aOR: 1,25 (95 % CI: 1,12-1,40)), pregabalin (aOR: 1,16 (95 % CI: 1,02-1,31)), oxcarbazepin (aOR: 1,48 (95 % CI: 1,28-1,71)), clonazepam (aOR: 1,27 (95 % CI: 1,10-1,48)) og topiramat ( aOR: 1,48 (95 % CI: 1,18-1,85)) forbundet med risiko for at blive født lille i forhold til svangerskabsalderen, og carbamazepin var forbundet med reduceret hovedomfang (mikrocefali) (aOR: 1,43 (95 % CI: 1,17-1,75)). Hos børn af mødre med epilepsi, var moderens brug i graviditeten af carbamazepin (aOR: 1,27 (95 % CI: 1,11-1,47)), oxcarbazepin (aOR: 1,42 (95 % CI: 1,18-1,70)), clonazepam (aOR: 1,40 (95 % CI: 1,03-1,89)) og topiramat (aOR: 1,86 (95 % CI: 1,36-2,54)) forbundet med at blive født lille i forhold til svangerskabsalderen og moderens brug af carbamazepin i graviditeten var forbundet med reduceret hovedomfang (mikrocefali) (aOR: 1,51 (95 % CI: 1,17-1,95)).

I studiet kunne man ikke påvise nogen sammenhænge mellem moderens brug af epilepsimedicin i graviditeten og risikoen for at blive født med lav fødselsvægt i forhold til svangerskabsalderen og reduceret hovedomfang (mikrocefali) for andre typer epilepsimedicin inklusiv lamotrigin, valproat, gabapentin, levetiracetam, phenobarbital, acetazolamid, phenytoin, clobazam, primidon, zonisamid, vigabatrin, ethosuximid og lacosoximid, men bortset fra lamotrigin, valproat, gabapentin og levetiracetam var antallet af børn i undersøgelsen lille.

**Konklusion:** Moderens brug i graviditeten af carbamazepin, oxcarbazepin, clonazepam og topiramat var forbundet med øget risiko for at blive født lille i forhold til svangerskabsalderen både i den samlede befolkning og blandt den grupper af børn der var født af mødre med epilepsi, hvilket tyder på, at udsættelse for disse typer epilepsimedicin i graviditeten er forbundet med påvirkning af fosterets vækst·

**Forskningsstøtte:** The NordForsk Nordic Program on Health and Welfare (83539)

# **Translated Abstract - Finnish abstract.**

The translations in Danish, Finnish, Icelandic, Norwegian, and Swedish were submitted by the authors, and we reproduce them as supplied. The translated abstracts have not been peer reviewed. Our editorial processes have only been applied to the original abstract in English, which should serve as reference for this manuscript.

**Yhteenveto**

**Taustaa:** Sikiön kasvuongelmien lyhyt- ja pitkäaikaiset seuraukset ovat merkittävä huolenaihe. Epäselvää on, miten äidin eri epilepsialääkkeiden käyttö raskauden aikana vaikuttaa sikiön kasvuun.

**Menetelmät:** Tässä väestöpohjaisessa tutkimuksessa olivat mukana Islannissa, Norjassa, Ruotsissa, Suomessa ja Tanskassa vuosina 1996–2017 elävänä syntyneet yksisikiöiset lapset. Epilepsialääkkeiden käyttö raskauden aikana määriteltiin kansallisten lääkerekistereiden ja äidin raskauden aikana tekemien reseptilääkeostojen perusteella. Tutkimuksen ensisijaiset tulosmittarit olivat vastasyntyneen alhainen raskauden kestoon suhteutettu syntymäpaino ja pieni päänympärysmitta (pienipäisyys eli mikrokefalia), joille laskettiin taustatekijöiden mukaan vakioidut vetosuhteet (aOR).

**Tulokset:** Tutkimuksessa oli mukana 4 494 920 lasta (poikia: 51,3 %), joista 38 710 (0,9 %) oli epilepsiadiagnoosin saaneiden äitien lapsia. Koko aineistossa äidin raskaudenaikainen karbamatsepiinin (aOR: 1,25, 95 %:n luottamusväli: 1,12–1,40), pregabaliinin (aOR: 1,16, 95 %:n luottamusväli 1,02–1,31), okskarbatsepiinin (aOR: 1,48, 95 %:n luottamusväli: 1,28–1,71), klonatsepaamin (aOR: 1,27, 95 %:n luottamusväli: 1,10–1,48) ja topiramaatin (aOR: 1,48, 95 %:n luottamusväli: 1,18–1,85) käyttö ainoana epilepsialääkkeenä liittyi alhaiseen raskauden kestoon suhteutetun syntymäpainon riskiin ja karbamatsepiinin käyttö liittyi pienen päänympärysmitan riskiin (aOR: 1,43, 95 %:n luottamusväli: 1,17–1,75). Epilepsiaa sairastavien äitien lapsilla karbamatsepiinin (aOR: 1,27, 95 %:n luottamusväli: 1,11–1,47), okskarbatsepiinin (aOR: 1,42, 95 %:n luottamusväli: 1,18–1,70), klonatsepaamin (aOR: 1,40, 95 %:n luottamusväli: 1,03–1,89) ja topiramaatin (aOR: 1,86, 95 %:n luottamusväli: 1,36–2,54) käyttö raskauden aikana liittyi alhaiseen raskauden kestoon suhteutettuun syntymäpainoon. Äidin raskaudenaikainen karbamatsepiiniin käyttö oli yhteydessä pieneen päänympärysmittaan (aOR: 1,51, 95 %:n luottamusväli: 1,17–1,95).

Tutkimuksessa ei voitu osoittaa yhteyttä äidin muiden epilepsialääkkeiden –lamotrigiini, valproaatti, gabapentiini, levetirasetaami, fenobarbitaali, asetsolamidi, fenytoiini, klobatsaami, primidoni, tsonisamidi, vigabatriini, etosuksimidi ja lakosamidi – raskaudenaikaisen käytön ja alhaisen raskauden kestoon suhteutetun syntymäpainon tai pienen päänympärysmitan välillä, mutta lamotrigiinia, valproaattia, gabapentiiniä ja levetirasetaamia lukuun ottamatta altistuneiden lasten määrä oli vähäinen.

**Johtopäätös:** Karbamatsepiinin, okskarbatsepiinin, klonatsepaamin ja topiramaatin käyttö raskauden aikana liittyi lisääntyneeseen alhaisen raskauden kestoon suhteutetun syntymäpainon riskiin koko väestössä ja epilepsiadiagnoosin saaneiden naisten lapsilla. Tulokset viittaavat siihen, että altistuminen näille epilepsialääkkeille on yhteydessä sikiön kasvuun.

**Tutkimusrahoitus:** NordForskin rahoittama Pohjoismainen terveyden ja hyvinvoinnin tutkimusohjelma (Nordic Project on Health and Welfare, hanke 83539).

#

# **Translated Abstract - Icelandic abstract.**

The translations in Danish, Finnish, Icelandic, Norwegian, and Swedish were submitted by the authors, and we reproduce them as supplied. The translated abstracts have not been peer reviewed. Our editorial processes have only been applied to the original abstract in English, which should serve as reference for this manuscript.

**Samantekt**

**Bakgrunnur:** Afleiðingar vaxtarskerðingar fósturs geta verið alvarlegar til skamms- og langs tíma. Óvíst hvort útsetning fyrir flogaveikislyfjum(ASM) í móðurkviði hefur áhrif á fósturvöxt.

**Aðferðir:** Rannsóknin var lýðgrunduð ferilrannsókn á lifandi fæddum einburum í Danmörku, Finnlandi, Íslandi, Noregi og Svíþjóð á árunum 1996 til 2017. Útsetning var skilgreind sem útleyst flogaveikislyf móður á meðgöngu samkvæmt lyfjagagnagrunn og aðalútkomur voru leiðrétt gagnlíkindahlutföll (aORs) fyrir ummerkjum um vaxtaskerðingu fósturs, mæld sem léttburafæðing (þyngd undir þyngd undir 10 og 3 percentili miðað við meðgöngulengd) eða lítið höfuðummál barns (höfuðsmæð).

**Niðurstöður:** Meðal 4.494.920 fæddra barna (drengir: 51.3%), voru 38.710 (0.9%) börn mæðra með flogaveiki. Í heildarþýðinu var útsetning á fósturskeiði fyrir einlyfjameðferð með karbamazepíni (aOR: 1.25 (95% CI: 1.12-1.40)), pregabalíni (aOR: 1.16 (95% CI: 1.02-1.31)), oxkarbazepíni (aOR: 1.48 (95% CI: 1.28-1.71)), klónazepami (aOR: 1.27 (95% CI: 1.10-1.48)) og tópíramati ( aOR: 1.48 (95% CI: 1.18-1.85)) tengd áhættu á léttburafæðingu og karbamazepín tengdist höfuðsmæð (aOR: 1.43 (95% CI: 1.17-1.75)). Hjá börnum mæðra með flogaveiki var útsetning á meðgöngu fyrir karbamazepíni (aOR: 1.27 (95% CI: 1.11-1.47)), oxcarbazepíni (aOR: 1.42 (95% CI: 1.18-1.70)), klónazepam (aOR: 1.40 (95% CI: 1.03-1.89)) og tópíramati (aOR: 1.86 (95% CI: 1.36-2.54)) tengd áhættu á léttburafæðingu; og karbamazepín tengdist höfuðsmæð (aOR: 1.51 (95% CI: 1.17-1.95)).

Útsetning á fósturskeiði fyrir lamótrigíni, valpróati, gabapentíni, levetiracetam, fenóbarbital, asetasólamíði, fenýtóíni, klóbazami, prímídóni, zonisamíði, vigabatríni, etosúxímíði og lakósímótíni virtist ekki auka áhættu léttburafæðingum eða og höfuðsmæð. En taka ber fram að fá börn í þýðinu voru útsett fyrir einlyfjameðferð með fenóbarbital, asetasólamíði, fenýtóíni, klóbazami, prímídóni, zonisamíði, vigabatríni, etosúxímíði og lakósímótíni.

**Túlkun:** Notkun á karbamazepíni, oxcarbazepíni, klónazepami og tópíramati á meðgöngu tengdist aukinni áhættu á léttburafæðingum og höfuðsmæð barns meðal kvenna með flogaveiki sem bendir til þess að útsetning fyrir þessum lyfjum á fósturskeiði tengist vaxtarskerðingu.

# **Translated Abstract - Norwegian abstract.**

The translations in Danish, Finnish, Icelandic, Norwegian, and Swedish were submitted by the authors, and we reproduce them as supplied. The translated abstracts have not been peer reviewed. Our editorial processes have only been applied to the original abstract in English, which should serve as reference for this manuscript.

**Sammendrag**

**Bakgrunn: Lav fødselsvekt kan ha konsekvenser for barnet både på kort og lang sikt. Det er usikkert om mors bruk av ulike epilepsimedisiner under graviditeten påvirker fosterets vekst.**

**Metode**:  **Dette er en undersøkelse av barn født i Danmark, Finland, Island, Norge og Sverige 1996 - 2017. Mors bruk av epilepsimedisin under graviditeten ble identifisert i nasjonale reseptregistre ut fra mors uthenting av resepter på slik medisin under graviditeten. Det primære resultatet i undersøkelsen var risikoen (odds ratio (OR)) for å bli født med lav fødselsvekt eller liten hodeomkrets.**

**Resultater:** I studien inngikk 4.494.920 barn (gutter: 51,3%), hvorav 38.710 (0,9%) barn ble født av mødre med epilepsi. I den samlede gruppen var mors bruk i graviditeten av carbamazepin (aOR: 1,25 (95 % CI: 1,12-1,40)), pregabalin (aOR: 1,16 (95 % CI: 1,02-1,31)), oxcarbazepin (aOR: 1,48 (95 % CI: 1,28-1,71)), clonazepam (aOR: 1,27 (95 % CI: 1,10-1,48)) og topiramat ( aOR: 1,48 (95 % CI: 1,18-1,85)) forbundet med økt risiko for lav fødselsvekt i forhold til svangerskapsalder· Carbamazepin var forbundet med liten hodeomkrets (mikrocefali) (aOR: 1,43 (95 % CI: 1,17-1,75)). Hos barn av mødre med epilepsi, var mors bruk i graviditeten av carbamazepin (aOR: 1,27 (95 % CI: 1,11-1,47)), oxcarbazepin (aOR: 1,42 (95 % CI: 1,18-1,70)), clonazepam (aOR: 1,40 (95 % CI: 1,03-1,89)) og topiramat (aOR: 1,86 (95 % CI: 1,36-2,54)) forbundet med lav fødselsvekt i forhold til svangerskapsalder. Mors bruk af carbamazepin i graviditeten var forbundet med liten hodeomkrets (mikrocefali) (aOR: 1,51 (95 % CI: 1,17-1,95)).

Vi kunne ikke påvise noen sammenheng mellem mors bruk i graviditeten og risikoen for lav fødselsvekt i forhold til svangerskapsalder eller liten hodeomkrets (mikrocefali) for andre typer epilepsimedisin, inkludert lamotrigin, valproat, gabapentin, levetiracetam, phenobarbital, acetazolamid, phenytoin, clobazam, primidon, zonisamid, vigabatrin, ethosuximid og lacosoximid. Utenom for lamotrigin, valproat, gabapentin og levetiracetam deltok det imidlertid et lavt antall barn i studien.

**Konklusjon:** Mors bruk i graviditeten av carbamazepin, oxcarbazepin, clonazepam og topiramat var assosiert med økt risiko for lav fødselsvekt i forhold til svangerskapsalder både i den samlete befolkningen og blant barn født av mødre med epilepsi. Dette tyder på at eksposisjon for flere typer epilepsimedisin i svangerskapet påvirker fosterets vekst.

**Forskningsstøtte:** The NordForsk Nordic Program on Health and Welfare (83539)

# **Translated Abstract - Swedish abstract.**

The translations in Danish, Finnish, Icelandic, Norwegian, and Swedish were submitted by the authors, and we reproduce them as supplied. The translated abstracts have not been peer reviewed. Our editorial processes have only been applied to the original abstract in English, which should serve as reference for this manuscript.

**Sammanfattning**

**Bakgrund: Låg födelsevikt kan ha såväl kort-som långsiktiga konsekvenser, och det är oklart hur moderns användning av olika epilepsiläkemedel under graviditeten påverkar fostrets tillväxt.**

**Metod**:  **Denna studie omfattar barn födda i Danmark, Finland, Island, Norge och Sverige från 1996 till 2017. Moderens användning av epilepsiläkemedel under graviditeten fastställdes genom nationella förskrivningsregister utifrån moderns uthämtande av recept under graviditeten. Studiens primära utfallsmått var risken (odds ration (OR)) för att födas med låg födelsevikt eller litet huvudomfång.**

**Resultat: I studien ingår** 4.494.920 barn (pojkar: 51,3%), varav 38.710 (0,9%) barn till mödrar med epilepsi. I den totala gruppen av barn var moderns användning under graviditeten av karbamazepin (aOR: 1,25 (95 % CI: 1,12-1,40)), pregabalin (aOR: 1,16 (95 % CI: 1,02-1,31)), oxkarbazepin (aOR: 1,48 (95 % CI: 1,28-1,71)), klonazepam (aOR: 1,27 (95 % CI: 1,10-1,48)) och topiramat ( aOR: 1,48 (95 % CI: 1,18-1,85)) associerad med risk för att födas liten för tiden, och karbamazepin associerad med litet huvudomfång (mikrocefali) (aOR: 1,43 (95 % CI: 1,17-1,75)). Hos barn till mödrar med epilepsi, var moderens användning under graviditeten av karbamazepin (aOR: 1,27 (95 % CI: 1,11-1,47)), oxkarbazepin (aOR: 1,42 (95 % CI: 1,18-1,70)), klonazepam (aOR: 1,40 (95 % CI: 1,03-1,89)) och topiramat (aOR: 1,86 (95 % CI: 1,36-2,54)) associerad med att födas liten för tiden och moderens användning av karbamazepin under graviditeten associerad med litet huvudomfång (mikrocefali) (aOR: 1,51 (95 % CI: 1,17-1,95)).

Studien kunde inte påvisa något samband mellan moderns användning av epilepsiläkemedel under graviditeten och risk för att födas liten för tiden eller med litet huvudomfång (mikrocefali) för andra typer av epilepsiläkemedel såsom lamotrigin, valproat, gabapentin, levetiracetam, fenobarbital, acetazolamid, fenytoin, clobazam, primidon, zonisamid, vigabatrin, ethosuximid och lakosamid, men bortsett från lamotrigin, valproat, gabapentin och levetiracetam var antallet exponerade barn litet.

**Konklusion:** Moderns användning under graviditeten av karbamazepin, oxkarbazepin, klonazepam och topiramat var associerad med förhöjd risk att födas liten för tiden såväl i den totala populationen som bland gruppen av barn till kvinnor med epilepsi, vilket tyder på att exponering för dessa typer av epilepsiläkemedel är förenad med påverkad fostertillväxt.

**Forskningsfinansiering:** The NordForsk Nordic Program on Health and Welfare (83539)
